# Prevalence of mental illness in primary care and its association with deprivation and social fragmentation at the small-area level in England

**DOI:** 10.1017/S0033291719000023

**Published:** 2019-02-12

**Authors:** Christos Grigoroglou, Luke Munford, Roger T. Webb, Nav Kapur, Darren M. Ashcroft, Evangelos Kontopantelis

**Affiliations:** 1 Division of Population Health, Health Services Research and Primary Care, NIHR School for Primary Care Research, Centre for Primary Care, University of Manchester, Manchester, UK; 2 Manchester Academic Health Sciences Centre (MAHSC), Manchester, UK; 3 Division of Population Health, Health Services Research and Primary Care, Centre for Health Economics, University of Manchester, Manchester, UK; 4 Division of Psychology and Mental Health, Centre for Mental Health and Safety, University of Manchester, Manchester, UK; 5 NIHR Greater Manchester Patient Safety Translational Research Centre, Manchester, UK; 6 Division of Psychology and Mental Health, Centre for Suicide Prevention, University of Manchester, Manchester, UK; 7 Greater Manchester Mental Health Trust; 8 Faculty of Biology, Medicine and Health, Centre for Pharmacoepidemiology and Drug Safety, School of Health Sciences, University of Manchester, Manchester, UK

**Keywords:** Antidepressant prescribing, depression, deprivation, mental illness, severe mental illness, social fragmentation

## Abstract

**Background:**

We aimed to spatially describe mental illness prevalence in England at small-area geographical level, as measured by prevalence of depression, severe mental illness (SMI) and antidepressant prescription volume in primary care records, and how much of their variation was explained by deprivation, social fragmentation and sociodemographic characteristics.

**Methods:**

Information on prevalence of depression and SMI was obtained from the Quality and Outcomes Framework (QOF) administrative dataset for 2015/16 and the national dispensing dataset for 2015/16. Linear regression models were fitted to examine ecological associations between deprivation, social fragmentation, other sociodemographic characteristics and mental illness prevalence.

**Results:**

Mental illness prevalence varied within and between regions, with clusters of high prevalence identified across England. Our models explained 33.4–68.2% of variability in prevalence, but substantial variability between regions remained after adjusting for covariates. People in socially cohesive and socially deprived areas were more likely to be diagnosed with depression, while people in more socially fragmented and more socially deprived areas were more likely to be diagnosed with SMI.

**Conclusions:**

Our findings suggest that to tackle mental health inequalities, attention needs to be targeted at more socially deprived localities. The role of social fragmentation warrants further investigation, and it is possible that depression remains undiagnosed in more socially fragmented areas. The wealth of routinely collected data can provide robust evidence to aid optimal resource allocation. If comparable data are available in other countries, similar methods could be deployed to identify high prevalence clusters and target funding to areas of greater need.

## Introduction

In developed countries such as the UK, most people diagnosed with mental illnesses receive their healthcare mainly via primary care (Cunningham, 2009; Care Quality Commission, 2015). In England, between 2013 and 2014, there were nearly 3 million adults on local general practice (GP) registers experiencing depression and approximately 500 000 were diagnosed with severe mental illness (SMI), which refers to patients with schizophrenia, bipolar disorder or other psychosis (Care Quality Commission, 2015). Moreover, prescribing of anti-depressants increased by 46% across England from 2012 to 2014 (NHS Digital, 2015).

Mental health problems demonstrate social gradients in the same way that physical health problems do, thereby indicating a stark health inequality (Marmot, 2005). Recent evidence from the UK suggests widening inequalities in mental health (Barr *et al*., 2015), with the national suicide rate increasing for over a decade and especially so in the most deprived areas (ONS, 2015). Many studies have emphasised that inequalities in mental health may be explained by neighbourhood or residential characteristics (Diez Roux and Mair, 2010). Living in a deprived or socioeconomically disadvantaged neighbourhood has been associated with poor health outcomes, including greater mortality, poorer self-reported health, adverse mental health outcomes and greater prevalence of chronic somatic disease (Diez Roux and Mair, 2010). In England, the largest increases in rates of suicide, self-reported mental health problems and antidepressant prescribing have been observed in the most deprived areas, leading to increasing inequalities in mental health (Delgadillo *et al*., 2016). Moreover, the quality of neighbourhood social capital and social cohesion may be particularly important to maintaining mental health independent of socioeconomic deprivation (Congdon, 1996; Ellen *et al*., 2001). Social fragmentation is often described as a measure of lack of social relationships within a geographical area and has been linked to mental disorders (Allardyce and Boydell, 2006; Fagg *et al*., 2006; Stafford *et al*., 2008) and SMI (Van Os *et al*., 2000; Richardson *et al*., 2018). More specifically, social fragmentation is often used to define neighbourhood-level conditions that may impair social relationships within a neighbourhood and also inhibit the levels of social cohesion and social capital available to residents (Ivory *et al*., 2011).

To tackle health inequalities, integrated system-wide approaches are required where primary care is at the forefront of prevention, early intervention and recovery (NHS England, 2016). In 2004, this move was reflected in the UK by the introduction of initiatives such as the Quality and Outcomes Framework (QOF), which aimed to improve the quality of primary care, reduce variation in quality across providers, and routinely manage and monitor people with chronic conditions (Roland, 2004). Management of people with SMI has been incentivised since the start of the scheme, although there have been several changes to the quality indicators over time. From 2006, the QOF also included indicators for depression, e.g. requiring practices to assess the severity of detected depression on diagnosis and after starting treatment. Since the introduction of the QOF in 2004, annually updated prevalence data have been available for numerous chronic conditions at the practice level, and these could provide more precise, timely and comprehensive information for determining healthcare need as they can be used to calculate crude prevalence for chronic diseases at small-area level.

This national study aimed to describe the overall mental illness prevalence in England, as measured using data derived from the 2015–2016 QOF, and to evaluate the association between deprivation and social fragmentation, and depression and SMI in England for 2015–2016 at small-area geographical level. More specifically, we aimed to: (a) estimate prevalence of depression and SMI at small-area level and then spatially describe variation in these two conditions across 10 English regions; (b) quantify and describe the variability and spatial autocorrelation of mental illness across English regions; (c) determine the extent to which deprivation and social fragmentation, two area-level factors known to have an adverse impact on mental health, are associated with prevalence of depression and SMI; and (d) establish whether population/locality characteristics such as age, sex, ethnicity and urbanicity/rurality drive variability in these associations across English regions.

## Methods

### Data sources

Our unit of analysis in the main models and all sensitivity analyses was the Lower Layer Super Output Area (LSOA). LSOAs are administrative units of geography with an average population of 1500 people, and there were 32 844 such units across England following the 2011 Census. Area-level deprivation was measured by the Index of Multiple Deprivation (IMD) 2015 (Communities and Local Government, 2015), which quantifies relative levels of deprivation for all LSOAs across seven domains: income, employment, education and skills, health and disability, crime, barriers to housing and services, and living environment. Social fragmentation was measured using the Index of Social Fragmentation (Congdon, 1996), which is a summary measure derived from 2011 Census data on single-person households, non-married adults, households in private renting and a measure of population transience. Data on both indices were complete for all LSOAs.

Mental illness was measured as QOF-recorded prevalence of depression and SMI in English primary care in 2015–2016 and we refer to these measures as prevalence of depression and SMI, respectively, throughout the manuscript. Under the QOF, recording for both conditions was incentivised for all 7619 participating GPs in 2015–2016 and we obtained data on disease prevalence and the respective practice registers from NHS Digital. QOF depression practice registers include only those 18 years old or older, whereas the SMI registers include the whole population which is registered on GPs and these registers were used as denominators for estimating data at the LSOA level. As a sensitivity analysis, we calculated antidepressant prescription volume comparators for each participating practice with data provided from NHS Digital. The volume comparators represent average daily quantities of antidepressants per specific therapeutic group age–sex-related prescribing units (NHS Digital). The volume comparators used in this instance compare drug prescribing between different practices and they are designed to weight individual practice or organisation populations for age and sex while taking into account the different needs of people who will be receiving that treatment. All three mental illness measures were attributed to LSOAs using spatial methodologies (Kontopantelis *et al*., 2015*b*). More information on the data and the attribution methodology are provided in the online Supplementary material.

We also obtained data from the 2011 decennial national Census at the LSOA level, directly (rural/urban classification and ethnicity for 2011) or derived (population estimates by gender and age group for 2015). To allow for comparisons within England, we organised LSOAs into 10 administrative regions, former SHAs (Strategic Health Authorities): North East, North West, Yorkshire and the Humber, East Midlands, West Midlands, East of England, London, South East Coast, South Central and South West.

### Analyses

The primary outcomes examined were recorded prevalence of SMI and depression in 2015–2016, and a measure of antidepressant prescription volume over the same annual period. The key covariates were the IMD 2015 and the Index of Social Fragmentation 2011, both measured at the LSOA level. We used digital mapping software to visualise the spatial distribution of the outcome variables across England and within regions. Box plots were produced to illustrate the distribution of recorded prevalence of depression and SMI as well as the distribution of antidepressant prescription volumes within each region.

We examined the extent to which spatial autocorrelation (i.e. correlation in a signal among nearby locations in space) was presented for the three outcomes examined. To assess the degree of global spatial autocorrelation, we calculated Moran's *I* (Moran, 1950). The measure can identify spatial clusters while accounting for the multi-dimensional and multi-directional nature of spatial autocorrelation. For example, Moran's *I* values close to unity for the prevalence of depression (high clustering) would indicate that areas with high levels of depression are clustered and also that LSOAs with high prevalence of depression are bordered with LSOAs with similarly high levels of depression. In turn, this would violate our assumptions about residual independence and affect the precision of our model estimates. We calculated Moran's *I* for each region and the whole of England to enable for within-England comparisons.

We fitted a set of linear regression models, weighted for 2015 LSOA population size, to quantify the association between each of the three outcomes of interest and deprivation, social fragmentation, demographic characteristics (age, sex and ethnicity), rurality and region (a detailed description of these variables is provided in the online Supplementary material). Variation at the regional level was quantified through this model with adjustment for other covariates. A second set of analyses was performed to explore interactions between rurality and social fragmentation and assess whether the examined associations vary across different residential settings such as large/small rural or urban areas. A third set of models were generated with interaction terms fitted between region and deprivation to assess whether the associations between deprivation and outcomes varied across regions. Similarly, a fourth set of models included interaction terms fitted between region and social fragmentation to investigate variations in the association between social fragmentation and outcomes across regions. The results from the second set of models are discussed in the main text, whereas the results from the third and fourth set of models as well as all sensitivity analyses are provided in the online Supplementary material. Stata v14.1 was used for the principal data management and analyses and R v3.3.1 was used to perform various spatial autocorrelation analyses. Although a two-sided *α* level of 5% was used throughout, the interpretation of *p*-values is not particularly meaningful in this context due to the size of the dataset (Lin *et al*., 2013), and we therefore focussed on the strength of the observed associations instead.

## Results

**Fig. 1.**
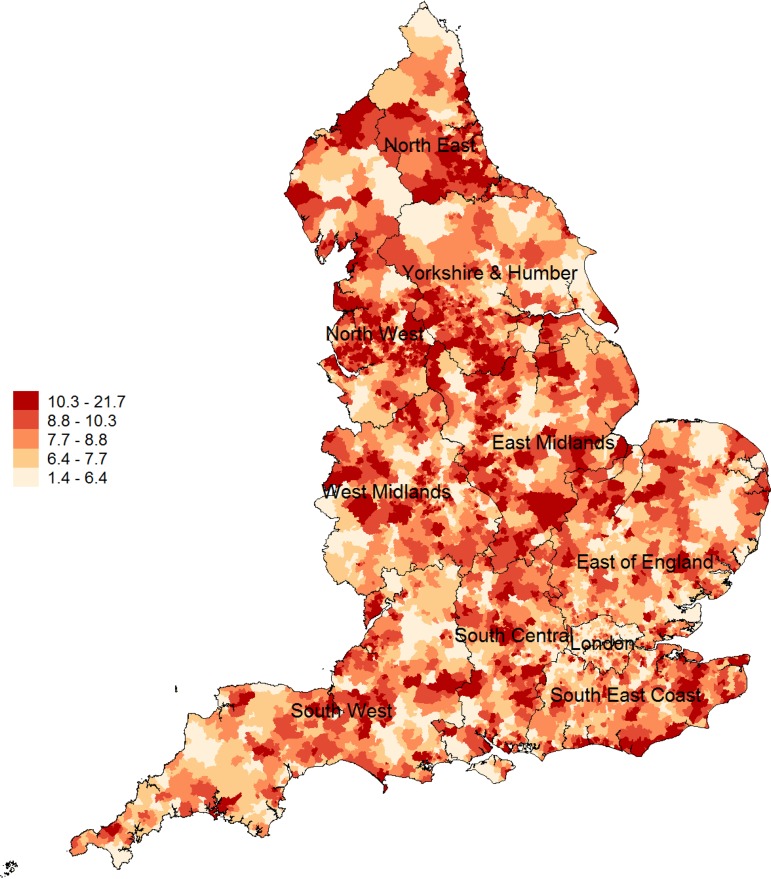
Prevalence of depression in England (2015/16 LSOA level).

Approximately 99% of the population of England was registered with a GP at the beginning of our study period in April 2015 (NHS Digital, 2015/16). Based on practice registers, mean % prevalence of depression, SMI and mean antidepressant prescription volume at the LSOA level for 2015–2016 were 8.38 (median: 8.21, IQR: 7.04–9.80), 0.89 (median: 0.85, IQR: 0.71–1.03) and 1.52 (median: 1.53, IQR: 1.21–1.84), respectively (Table 1, Fig. 1A in the online Supplementary material). Mean % prevalence of depression varied from 6.10 (median: 6.04, IQR: 5.03–7.07) in London to 9.68 (median: 9.54, IQR: 8.06–11.2) in the North West. Mean % prevalence of SMI varied from 0.78 (median: 0.75, IQR: 0.63–0.89) in South Central to 1.08 (median: 1.03, IQR: 0.84–1.27) in London. Mean antidepressant prescription volume varied from 0.87 (median: 0.88, IQR: 0.74–1.00) in London to 2.26 (median: 2.27, IQR: 2.02–2.44) in the North East.

**Table 1.** Characteristics at a small-area geographical area (LSOA) across England and each of its 10 regions^a^

	England	North East	North West	Yorkshire & the Humber	East Midlands	West Midlands	East of England	London	South East	South Central	South West
Aggregates across LSOAs											
Number of LSOAs	32 844	1657	4497	3317	2774	3487	3614	4835	2773	2609	3281
Total population	54 786 324	2 624 619	7 173 835	5 390 576	4 677 038	5 751 000	6 076 451	8 673 313	4 635 616	4 312 297	5 471 180
%Rural	17.0	17.6	9.8	16.5	25.4	14.8	28.3	0.2	20.1	19.8	30.2
Means (25^th^ and 75^th^ centiles) across LSOAs											
Prevalence of depression	8.38 (7.04, 9.80)	9.16 (7.62, 10.49)	9.68 (8.06, 11.2)	8.74 (7.30, 9.91)	9.19 (7.55, 10.8)	8.64 (7.05, 9.96)	8.02 (6.77, 9.20)	6.10 (5.03, 7.07)	8.54 (7.21, 9.85)	8.39 (6.82, 9.68)	8.46 (7.03, 9.80)
Prevalence of severe mental illness	0.89 (0.71, 0.91)	0.91 (0.81, 1.00)	1.00 (0.82, 1.14)	0.85 (0.70, 0.98)	0.81 (0.65, 0.95)	0.85 (0.69, 0.98)	082 (0.66, 0.93)	1.08 (0.84, 1.27)	0.82 (0.65, 0.93)	0.78 (0.63, 0.89)	0.83 (0.67, 0.95)
Antidepressant prescriptions volume (ADQ/STARPU)	1.52 (1.21, 1.84)	2.26 (2.02, 2.44)	1.98 (1.70, 2.21)	1.71 (1.49, 1.91)	1.60 (1.38, 1.78)	1.46 (1.29, 1.61)	1.51 (1.22, 1.79)	0.87 (0.74, 1.00)	1.42 (1.21, 1.60)	1.48 (1.17, 1.74)	1.57 (1.39, 1.70)
Medians (25^th^ and 75^th^ centiles) across LSOAs											
Depression cases (*N*)	14 (11, 18)	138 (115, 166)	148 (121, 180)	13 (11, 16)	148 (120, 183)	13 (11, 17)	128 (105, 156)	105 (86, 127)	12 (10, 16)	12 (10, 15)	13 (10, 16)
Severe mental illness cases (*N*)	132 (107, 163)	14 (12, 16)	15 (12, 19)	136 (112, 165)	13 (10, 16)	134 (111, 164)	13 (10, 16)	18 (14, 23)	138 (112. 167)	131 (108, 158)	135 (111, 164)
Index of social fragmentation^b^	−0.70 (−2.29, 1.43)	−0.59 (−2.16, 0.97)	−0.51 (−2.32, 1.62)	−0.78 (−2.30, 1.07)	−1.27 (−2.65, 0.58)	−1.01 (−2.45, 0.65)	−1.30 (−2.59, 0.40)	1.58 (−0.64, 3.98)	−1.20 (−2.50, 0.76)	−1.27 (−2.63, 0.62)	−0.87 (−2.17, 1.02)
IMD^c^ ^(continuous measure)^	17.3 ( 9.65, 30.1)	23.2 (11.6, 38.2)	21.6 (10.9, 39.5)	19.8 (11.0, 36.9)	16.4 ( 9.2, 29.0)	20.2 (11.36, 36.0)	14.5 ( 8.3, 22.9)	22.1 (13.3, 32.7)	12.7 (7.3, 20.9)	10.7 ( 5.7, 19.3)	15.2 ( 9.3, 23.4)
%Female	50.9 (49.8, 52.1)	51.3 (50.0, 52.4)	51.0 (49.8, 52.1)	51.1 (49.9, 52.2)	50.9 (49.9, 51.9)	50.8 (49.7, 51.8)	51.0 (49.9, 52.0)	50.7 (49., 52.0)	51.2 (50.1, 52.3)	50.8 (49.7, 52.0)	51.2 (50.0, 52.2)
%Aged 25–44 (of age 18 + )	25.2 (20.8, 30.0)	23.9 (20.9, 26.5)	24.7 (20.9, 28.2)	24.5 (20.9, 28.3)	24.0 (20.1, 27.7)	24.9 (20.9, 28.5)	24.4 (20.2, 29.0)	33.4 (28.5, 40.0)	23.3 (19.3, 27.8)	25.0 (20.5, 30.0)	22.4 (18.3, 26.9)
% Aged 45–64 (of age 18 + )	26.3 (22.8, 29.3)	27.6 (25.1, 30.4)	26.9 (23.8, 29.6)	26.8 (23.3, 29.8)	27.2 (24.0, 30.2)	25.9 (22.6, 28.9)	26.6 (23.6, 29.3)	22.4 (19.6, 25.1)	26.9 (24.3, 29.5)	26.8 (23.1, 29.7)	27.2 (24.3, 30.1)
% Aged 65 or over (of age 18 + )	17.7 (12.2, 23.5)	18.9 (14.6, 23.9)	18.0 (13.3, 23.6)	18.6 (13.3, 23.6)	18.3 (13.3, 24.7)	18.1 (13.1, 24.0)	19.2 (13.8, 24.7)	11.0 (8.14, 14.8)	19.6 (14.8, 24.8)	17.7 (12.0, 23.3)	21.7 (15.4, 27.3)
%White British	94.8 (83.3, 97.7)	98.0 (95.8, 98.8)	96.5 (91.9, 98.1)	96.6 (90.4, 98.2)	96.7 (89.6, 98.1)	92.8 (79.3, 97.4)	94.8 (89.2, 97.4)	63.1 (45.9, 77.6)	94.8 (90.4, 97.1)	93.9 (86.4, 96.8)	97.6 (95.2, 98.6)

a Variables in general reported for 2015 calendar year, except:, fragmentation (2011 Census), ethnicity (2011 Census).

b Index of Social Fragmentation, composite measure constructed based on 2011 census data, numbers closer to +ve indicate more socially cohesive areas.

c Index of Multiple Deprivation, details available in the 2015 technical report of the English Indices of Deprivation.

**Fig. 2.**
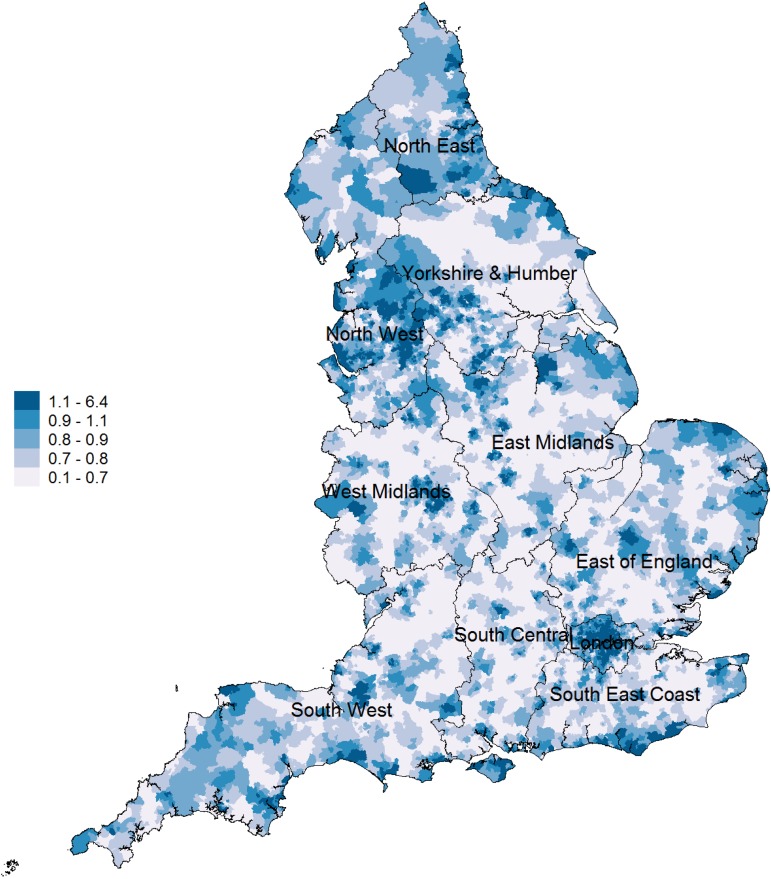
Prevalence of severe mental illness in England (2015/16 LSOA level).

We present spatial variability at the LSOA level for the three outcome measures in Figs 1 and 2 and Fig. 3A (online Supplementary material). Antidepressant prescription volume was strongly correlated with QOF recorded prevalence of depression (Pearson *ρ* was 0.58). We looked at the regional variability for the three outcomes of interest and we generated spatial maps which are provided in the online Supplementary material. We observed great variability for all three outcomes across and within regions. High prevalence of depression was concentrated in the North West, South East and South West. London had the lowest levels of depression followed by the East of England and the North East. There was a clear distinction between rural and urban areas, with the exception of London. Urban areas appeared to have greater recorded levels of depression. For SMI, we also observed great variability across and within regions. We observed similar patterns for depression and SMI, with the exception of London. For example, increased levels of SMI were observed in urban areas in the South East and North West regions, but London had by far the greatest recorded levels for SMI across regions. High volume of antidepressant prescription was concentrated in the North East, North West and West Midlands. The lowest levels of antidepressant prescription volume were observed in the South West and South Central, while we observed similar patterns to prevalence of depression in some areas but not in others.

### Results from analysis, adjusted associations at the LSOA level

At the LSOA level, after adjusting for age, sex, rurality, social fragmentation and deprivation, large variability between regions persisted, for the three outcomes (Tables 2 and 3 and Table A3 in online Supplementary material). For depression, the North West had 6.5% (95% CI 5.6–7.4%) higher mean prevalence of depression than the North East (reference region). Social fragmentation was a strong predictor for prevalence of depression, but the association observed was in the opposite direction to that we expected to find. A one-unit increase in social fragmentation, e.g. from −0.701 (50^th^ centile) to 0.299 (64^th^ centile) was associated with a 7.6% decrease in depression prevalence within an LSOA. For IMD, a 10-unit increase in the composite measure, e.g. from 17.4 (50^th^ centile) to 27.4 (71^th^ centile) corresponded to a 4% increase in depression prevalence. Urbanicity was also a strong predictor, with rural areas associated with 0.76% (95% CI 0.65–0.82%) lower prevalence of depression. Depression was also more prevalent in LSOAs with a large population in the 25–44 age group. A 10% increase in patients 25–44 years old in residents within an LSOA [e.g. from 25.1% (50^th^ centile) to 35.1 (89^th^ centile)] was associated with a 3.7% (95% CI 3.3–4.2%) increase in the prevalence of depression. For antidepressant prescription volumes, there was a broad agreement with the model for prevalence of depression. We present and discuss the results from the analysis with antidepressant prescription volume as the outcome variable in the online Supplementary material. Moreover, for the second set of models with the interaction term between rurality and social fragmentation, we found that recorded depression prevalence was higher in socially cohesive rural areas, socially cohesive minor urban areas and small cities, while socially fragmented urban areas had the lowest levels of recorded depression/antidepressant prescription volume.

**Table 2.** Results from model for depression, linear regression at the LSOA level^a^^,^^b^^,^^c^

Outcome: prevalence of depression	Coefficient^c^	95% Confidence interval		*p*-value
Region				
North East	Reference			
North West	0.654	0.544	0.764	0.001
Yorkshire and Humber	−0.076	−0.191	0.038	0.192
East Midlands	0.561	0.442	0.680	0.001
West Midlands	0.015	−0.099	0.130	0.781
East of England	−0.516	−0.630	−0.401	0.001
London	−1.720	−1.843	−1.597	0.001
South East	−0.011	−0.130	0.107	0.849
South Central	−0.017	−0.139	0.104	0.778
South West	−0.184	−0.300	−0.069	0.002
Demographics				
% aged 25–44, 2015	0.037	0.033	0.042	0.001
% aged 45–64, 2015	0.026	0.019	0.033	0.001
% aged 65 or over, 2015	−0.014	−0.018	−0.010	0.001
% female, 2015	0.011	0.002	0.021	0.016
% white British, 2015	0.045	0.043	0.046	0.001
LSOA metrics				
Rural LSOA	−0.758	−0.819	−0.696	0.001
Social fragmentation	−0.077	−0.086	−0.067	0.001
IMD 2015	0.040	0.039	0.042	0.001
Constant	1.942	1.382	2.502	0.001

a A total of 32 844 LSOAs (observations) with analytic weighting.

b Adjusted *R*^2^ = 33.39%.

c Coefficients can be interpreted as percentage change, for example, adjusted depression levels in the North West were 0.654% higher than in the North East.

**Table 3.** Results from model for SMI, linear regression at the LSOA level^a^^,^^b^^,^^c^

Outcome: prevalence of SMI	Coefficient^c^	95% Confidence interval		*p*-value
Region				
North East	Reference			
North West	0.064	0.053	0.076	0.001
Yorkshire and Humber	−0.069	−0.081	−0.057	0.001
East Midlands	−0.068	−0.080	−0.055	0.001
West Midlands	−0.065	−0.077	−0.053	0.001
East of England	−0.029	−0.041	−0.017	0.001
London	0.019	0.006	0.032	0.004
South East	−0.023	−0.035	−0.010	0.001
South Central	−0.058	−0.071	−0.045	0.001
South West	−0.021	−0.034	−0.009	0.001
Demographics				
% aged 25–44, 2015	0.007	0.007	0.008	0.001
% aged 45–64, 2015	0.012	0.011	0.013	0.001
% aged 65 plus, 2015	0.006	0.006	0.007	0.001
% female, 2015	0.004	0.003	0.005	0.001
% white British, 2015	−0.003	−0.003	−0.003	0.001
LSOA metrics				
Rural LSOA	−0.114	−0.120	−0.107	0.001
Social Fragmentation	0.022	0.021	0.023	0.001
IMD 2015	0.005	0.005	0.006	0.001
Constant	0.263	0.203	0.322	0.001

a A total of 32 844 LSOAs (observations) with analytic weighting.

b Adjusted *R*^2^ = 44.42%.

c Coefficients can be interpreted as percentage change, for example, adjusted SMI levels in the North West were 0.064% higher than in the North East.

For SMI, when compared with the North East (reference region), the North West had 6.4% (95% CI 5.3–7.6%) higher mean prevalence of SMI. Social fragmentation was a strong predictor for prevalence of SMI, with a one-unit increase in social fragmentation, e.g. from −0.701 (50^th^ centile) to 0.299 (64^th^ centile) associated with a 2.2% increase in SMI prevalence within an LSOA. For the IMD, a 10-unit increase in the IMD, e.g. from 17.4 (50^th^ centile) to 27.4 (71^th^ centile) was associated with 0.5% increase in the prevalence of SMI within an LSOA. Similar to depression, urbanicity was also a strong predictor, with rural areas associated with 0.11% (95% CI 0.10–0.12%) lower prevalence of SMI. The highest prevalence of SMI was observed for LSOAs with a higher proportion of the 45–64 age group. A 10% increase in residents in the 45–64 range within an LSOA [e.g. from 22.81% (25^th^ centile) to 32.81% (93^th^ centile)] was associated with a 1.2% (95% CI 1.1–1.3%) increase in the prevalence of SMI.

We also present spatial autocorrelation for prevalence of depression, SMI and antidepressant prescription volume across regions in Fig. 2A in the online Supplementary material. For depression, the global Moran's *I* index showed significant positive spatial autocorrelation (0.176; *p* < 0.001) for the whole of England, evidencing the existence of low spatial dependence among LSOAs with similar patterns of prevalence of depression. At the regional level, we observed moderate variability in spatial autocorrelation, with the lowest value observed in London (0.108; *p* < 0.001) and the highest in the South East region (0.294; *p* < 0.001). This indicates that areas with similar levels of depression in London are least clustered, whereas areas in the South East exhibit the greatest spatial autocorrelation. For SMI, global Moran's *I* index showed significant positive spatial autocorrelation (0.131; *p* < 0.001), again evidencing the presence of low spatial dependence among LSOAs with similar levels of prevalence of SMI. At the regional level, variability in spatial autocorrelation was more pronounced, ranging from 0.138 (*p* < 0.001) in the North East to 0.367 (*p* < 0.001) in the South East. For antidepressant prescription volume, the global Moran's *I* index showed the greatest positive spatial autocorrelation 0.394 (*p* < 0.001), evidencing moderate dependence among LSOAs with similar volumes of antidepressant prescription. At the regional level, variability for antidepressant prescription was greater, ranging from 0.112 (*p* < 0.001) in London to 0.545 (*p* < 000.1) in South Central.

## Discussion

This study provides evidence of marked heterogeneity in prevalence of depression and SMI both within and between regions. We identified large clusters of high mental illness prevalence across England and we also identified and quantified the associations of our three outcome variables with deprivation, social fragmentation and demographic characteristics. The two variables of primary interest, social fragmentation and deprivation, appeared to be strong predictors of mental illness prevalence even though social fragmentation was linked to lower depression prevalence. Our results also indicate that prevalence of both depression and SMI and antidepressant prescription volume are lower in rural areas.

Overall, spatial autocorrelation of the three outcome measures for the whole of England was low and we therefore did not account for spatial autocorrelation in our models. Regionally, the South East had moderate levels of spatial autocorrelation for prevalence of depression and SMI, while the South Central and East of England had high levels of spatial autocorrelation for antidepressant prescription. The regression models explained a reasonable level of variability for depression (33.4%), SMI (44.4%) and antidepressant prescription volume (68.2%) but large variability both within and between regions persisted, even after adjusting for covariates. Rurality was also a strong predictor, with lower levels for the outcomes examined observed in rural areas.

## Strengths and limitations

This study was conducted with a large, routinely collected database containing aggregated data on diagnoses of depression and SMI, as well as antidepressant prescribing, for a national population of over 55 million people. We generated population-wide maps of disease burden and we examined associations with deprivation and social fragmentation at an ecological level. Analysis at small-area level enabled us to associate mental illness prevalence with population characteristics such as deprivation and social fragmentation. Moreover, we could visualise the parameters of interest and identify spatial clusters of high and low need for relatively homogeneous populations.

The study also has a number of limitations. First, this is an ecological study conducted at small-area level and the possibility of ecological fallacy cannot be ruled out as we assigned practice-level information to small-area localities and we cannot determine how much of the ecological association is explained by variations in the distribution of individual-level risk factors. However, in the absence of individual-level data, the QOF is the primary source of information for prevalence of depression and SMI at the GP level and thus it allows estimation of data at a very low geographical level. This is important for targeting areas within regions that have substantially high prevalence of depression and SMI. However, should national individual-level data become available, an alternative approach would warrant the use of multilevel modelling methods. A second limitation is the use of 2011 Census data in the Index of Social Fragmentation and ethnicity variables as that was the latest available Census. Census data are collected every 10 years and the study period was in the middle of the Census decade, although the majority of LSOAs are known to change very little in terms of their sociodemographic profiles between the 10-year Censuses. Therefore, because the sociodemographic composition of virtually all LSOAs does not change that much between Censuses, we assumed that there has been little change over time in the data used for the social fragmentation index across regions and LSOAs, although this may not be the case. Third, QOF-recorded depression prevalence may be lower than the actual prevalence of depression in the population (Rait *et al*., 2009), as some GPs may prefer to record symptoms rather than formal psychiatric diagnoses (Kendrick *et al*., 2015). Thus, we calculated antidepressant prescription volumes at the LSOA level as an additional proxy measure for prevalence of depression in the population, with similar regression results observed to the analysis for prevalence of depression. Fourth, our estimates may have been affected by the choice of standard regression models over spatial autoregressive models, although weighing for LSOA size would not be possible under different assumptions. However, the overall level of spatial autocorrelation for the whole of England was low for prevalence of depression and SMI, and we trust that this has not affected the precision of our model estimates. Fifth, mood disorder, including depression, is used as an underlying indicator in the ‘health and deprivation’ domain of the IMD, although its weighting in the overall score is low, while the information on mood disorders is historical in relation to the data analysed.

## Interpretation of the findings

The regression models generated indicated a significant positive association of deprivation with the three outcome variables examined and there may be several reasons for this phenomenon. In England, the 2008 recession led to detrimental effects on mental health, especially for those areas that experienced the greatest rises in unemployment (Barr *et al*., 2012; Gunnell *et al.*, 2015). It is possible that austerity policies implemented after the recession have impacted adversely on mental health with some groups affected more than others (Barr *et al*., 2012) especially in more deprived areas (Iacobucci, 2014). Moreover, cuts to local government budget and the benefit reforms have hit the most deprived and poorest parts of the country hardest, leading to widening health inequalities (Taylor-Robinson and Gosling, 2011; Crawford and Phillips, 2012; Barr *et al*., 2016). Finally, it is suggested that recording of SMI in primary care exhibits a greater increase in the most deprived areas (Kontopantelis *et al*., 2015*a*).

Our models also indicated an unexpected negative association for prevalence of depression and antidepressant prescription volume with social fragmentation and we explored this association across different residential settings to conclude that there may be several reasons for the unexpected association observed. First, it may be that under-diagnosis is more common in socially fragmented areas and case finding and recording are worst in less fragmented areas. This is particularly relevant for rural areas and small cities, which are known to have higher rates of GP attendance for mental health problems and more specifically depression, when compared with urban areas (Perkins *et al*., 2013). Furthermore, highly socially fragmented/urban areas are mainly inhabited by young people (Thomas *et al*., 2015) who also live alone and are most likely to be private renters (Dorling *et al*., 2008) and several studies report that people who reside in urban centres, especially young people, may have negative perceptions about the value of consulting GPs for depressive symptoms (Biddle *et al*., 2006; Probst *et al*., 2006; Mauerhofer *et al*., 2009; Davey *et al*., 2013).

For SMI, we observed a modest association with social fragmentation, while the limited evidence that exists suggests mixed results and an unknown mechanism that drives the association (Allardyce *et al*., 2005; Kirkbride *et al*., 2014). However, the prevalence of SMI is elevated in urban areas (i.e. more socially fragmented) and urban residents may be more prone to develop SMI (Gruebner *et al*., 2017; Newbury *et al*., 2017).

We also found lower levels of mental illness in rural areas, which indicates that people in rural areas may experience more favourable psychosocial outcomes (e.g. less stress) in comparison to people living in urban areas and this could relate to the absence of urban environmental risk factors (Haynes and Gale, 1999). This finding may also indicate differences in the availability of mental health services between urban and rural areas, which may even lead to relocation to urban areas for access to mental health services (Wang, 2004). This finding, the differences in prevalence of common health conditions between urban and rural residents, is consistent with previous research on depression (Paykel *et al*., 2000; Weich *et al*., 2006; Riva *et al*., 2009).

Our small-area geographical mapping approach facilitates within-region investigations and the identification of geographical clusters of high disease prevalence. Large clusters of high prevalence of depression were observed in the North East, London and Yorkshire and Humber, and additional resources may need to be allocated to those areas that serve these populations (e.g. extended opening hours, re-distribution of GPs). Similarly, we identified large clusters of high prevalence of SMI in the South East, South Central and London. Even though the problem with increased levels of SMI in London was known (White *et al*., 2014), our results show that the problem persists in other areas as well. We observed high levels of spatial variation in prevalence of depression across the whole region in the North West and South East, which could also inform organisation of care to these areas. For SMI, with the exception of London, high levels of spatial variation in prevalence of SMI across the whole region were observed for the North West and South East.

The North West and North East had the largest within-region spatial variation in antidepressant prescription volume. In some areas, we observed opposite patterns for prevalence of depression and antidepressant prescription volume indicating that in areas where antidepressant prescription increased, prevalence of depression could be lower. This may be attributed to a combination of factors such as the documented over-prescription of antidepressants in England (NHS Digital, 2016), the antidepressant prescription habits of GPs for off-label indications (Wong *et al*., 2017) or the use of symptom depression codes rather than depression diagnoses in the recording of depression (Rait *et al*., 2009). We also observed higher prevalence of SMI in areas with high suicide rates (ONS, 2015), such as areas in the North East, South Central and South West, as expected. However, it is unclear whether these pathways persisted over time and further work is needed to investigate regional changes in prevalence of depression and SMI retrospectively.

## Conclusion

Much of the provision of frontline and follow-up services for patients with mental health problems is moving towards primary and community care, and at the same time, newly implemented health policies may contribute to the overarching purpose of achieving equitable healthcare. For instance, the Five Year Forward View plan predicts the expansion of primary care staff with investment in an extra 3000 mental health therapists to work in primary care by 2020 (NHS England, 2016), although this seems extremely optimistic (Esmail *et al*., 2017). Similar initiatives may prove to be highly beneficial in tackling inequalities in mental health, but it is also important to pay special attention to the most deprived and most socially fragmented areas, since deprivation and social fragmentation appear to be strongly associated with mental illness prevalence. Re-organisation of care to that extent will also require accounting for the increased demand for better mental health services in those areas with the greatest burden of mental disorder. The spatial nature of deprivation and social fragmentation can provide a wealth of information for effective organisation of health care and in conjunction with the retention of routinely collected data, such as QOF disease registers, may inform optimal allocation of resources.

## Supplementary material

For supplementary material accompanying this paper visit https://doi.org/10.1017/S0033291719000023. click here to view supplementary material
