# Supplementary material for: Prevalence of mental illness in primary care and its association with deprivation and social fragmentation at the small-area level in England
Source: Psychol Med. 2019 Feb 12;50(2):293–302. doi: 10.1017/S0033291719000023 (PMC7083582; doi:10.1017/S0033291719000023)
Supplement: Supplementary file 1 [file S0033291719000023sup001.docx]

**Supplementary material**

#

# Prevalence of mental illness in primary care and its association with deprivation and social fragmentation at small-area level: a spatial analysis in England

# Online appendix 1: details on methods and additional analyses

# Data collection

## English Indices of Deprivation

Area deprivation, as measured by the latest update of the Index of Multiple Deprivation (IMD) (i.e.2015) was available at the 2011 Lower Super Output (LSOA) level. The IMD is the most complete and widely used approach to quantify relative deprivation and affluence for small areas in England. It is a combined score of deprivation based on a total of 37 separate indicators that have been grouped into [seven domains](https://factsandfigures.herefordshire.gov.uk/about-a-topic/inequalities-and-deprivation/index-of-multiple-deprivation.aspx#Domains), each of which reflects a different aspect of deprivation experienced by individuals living in an area. The overall measure is calculated as a weighted mean across seven domains: income, employment, education and skills, health and disability, crime, barriers to housing and services, and living environment with different weights given to each domain. The Index of Multiple Deprivation is assigned to every small area in England and ranks them from 1 (most deprived area) to 32,844 (least deprived area). The IMD is widely used across central government to focus programmes on the most deprived areas. (Department of Communities and Local Government, 2015)

## Lower Super Output Areas

The low geographical units to which the indices of deprivation are assigned are called lower super output areas (LSOAs) and they are designed to contain around 1500 inhabitants, on average. Following the 2011 census, there were 32,844 English LSOAs. (ONS, 2012) Urbanity information was also updated following the census, and we used a rural vs urban dichotomy for simplicity, with settlements with 10,000 people or more defined as urban. (Bibby P) Census-adjusted population estimates over time, by age groups and sex and for each English LSOA, were obtained from the Office of National Statistics. (ONS, 2015) Spatial coordinates for the 2011 LSOAs were obtained from the ONS open geography portal. We used digital vector boundaries generalised to 20 meters and clipped to the coastline to reduce size and improve visualisation. (ONS, 2013b) To allow for comparisons within England, we organised LSOAs into 10 regions: North East, North West, Yorkshire & the Humber, East Midlands, West Midlands, East of England, London, South East Coast, South Central and South West.

## Index of Social Fragmentation

In the UK, an index of social fragmentation was developed to define anomic areas, which have been referred to as an index of social fragmentation, measuring lack of opportunities for social integration. (Congdon, 1996)  The index of social fragmentation has been used in ecological studies to analyse whether material deprivation and social fragmentation affect different types of diseases, and to establish whether it is possible to disentangle the effects of the two factors. We constructed an index of social fragmentation which is known to be associated with poor mental health. The summary index represents neighborhood-level conditions which affect social resources such as social cohesion and social capital. Congdon’s index is derived from census and migration data at the LSOA level from the Nomis Official Labour Market Statistics website (ONS, 2013a) on: (1) One person households, %of all 2011 census households; (2) unmarried people, % all 2011 census population; (3) Population turnover (percentage of the population that moved from other area or within the country), % population (4) Households in private renting, %of all 2011 census households. The social fragmentation index for each LSOA was calculated by adding the z scores (the number of standard deviations above or below the population mean when the underlying distribution is normal) for each of the four characteristics and the final score was re-standardised. The scores for the index of social fragmentation 2011 ranged from -7·09 to 25·79 across LSOAs and were only moderately correlated with the IMD scores (Pearson’s rho=0·41).

## Anti-depressant prescribing data

We also obtained quarterly data on anti-depressant prescription from NHS Digital. (NHS Digital, 2016) We used the prescription data as an additional measure of depression because recording of depression in English primary care is considered problematic. For each antidepressant, we calculated its ADQ/STARPU value in each year at the LSOA level. ADQ/STARPUs are drug volume prescription comparators between general practices and were primarily developed to highlight variation in prescribing across organisations.

For the definition of Average Daily Quantities (ADQ) values five principles have been considered. First, the ADQs are based on Defined Daily Dose (DDD) values which have been defined by the World Health Organisation (WHO); hence when ADQ values were not available their respective DDD values were used in the calculation of ADQ/STARPU. Second, the ADQ values also consider the Prescribed Daily Dose in conjunction with the DDD, as it reflects the actual usage by GPs. Third, prescription services data are taken into account to reflect the number of items prescribed by particular quantities of each drug preparation. Four, BNF information regarding dosage is sought. Five, the development of ADQ considers the therapeutic equivalency between drugs of the same therapeutic type. Due to the different indications for drugs and different prescribing habits in England, ADQs are preferred to DDDs and they are reviewed on a regular basis to reflect drug utilization and the introduction of new drugs. The ADQ values for the anti-depressants (BNF section 4.3) were obtained via NHS Digital (NHS Digital, 2016 ) and the latest update was accessed. Total ADQs for all antidepressants in our list were calculated using the formula. We added together the number of ADQs of all the drugs in the same broad therapeutic class or of all the drugs (i.e. antidepressant) and we obtained the total number of ADQs for antidepressants prescribed in one period for each practice. Subsequently we calculated an average ADQ/STARPU across the 12 periods.

Furthermore, we obtained data on specific therapeutic group age-sex related prescribing units (STAR-Pus) which are designed to weight individual practice populations for age, sex and temporary residents and they are therefore the appropriate denominators for cost of prescribing comparisons between general practices. STAR-Pus are available for the anti-depressants group and they have been developed according to the respective ADQ values for that group. The ADQ/STARPUs were calculated separately for all age groups and both genders. Finally, we calculated annual median ADQ/STARPUs for each general practice to account of the presence of ADQ/STARPU outliers. Regarding the list of antidepressants, we included all relevant drugs that are included in the BNF section 4.3 except for lithium as it is used to enhance the use of anti-depressants and was not considered appropriate for inclusion. A STAR-PU (or Specific Therapeutic group Age-sex Related Prescribing Unit) is a value calculated to reflect not only the number of patients in a practice, but also the age and sex mix of that group.

## Age groups

To adjust for population structure we obtained population estimates for 2015-2016 at the LSOA level, broken down by age group from the Office for National Statistics. (Statistics., 2015) We aggregated these population estimates in three larger age groups ((25-44, 45-64, 65+ (20-24 age group used as the reference group)), according to ONS classification, to address any issues of collinearity between age groups. We chose to aggregate population estimates in these specific age groups as these lower and upper bounds are used in similar analyses investigating place effects on depression and SMI. We also did various checks for collinearity between the three age groups and we did not observe a value >4 for the variance inflation factor across all models.

## Ethnicity

Ethnicity was taken from the 2011 census and was available at the LSOA level from the Nomis website. (ONS, 2013a) Ethnicity classifies people according to their own perceived ethnic group and cultural background. The variable uses a harmonised country specific ethnic group question and the question is recommended when a show card is used in a face to face interview or self-completion survey (both paper and electronic).

## Sex and population estimates

From the Office for National Statistics, we obtained mid-year population estimates for 2015 at the LSOA level. (ONS, 2015) The mid-year population estimates are the official set of population estimates for the UK and its constituent countries, the regions of England and Wales and local authorities. They are used directly as a base for other secondary population statistics, such as population projections, population estimates for the very old and population estimates for small geographical areas. A combination of registration, survey and administrative data are used to estimate the different components of population change. The data are provided for the whole population as well as by sex. We used the number of females in an LSOA in 2015 as recorded by ONS to calculate the percentage of females who were resident in each LSOA in 2015-2016.

## Prevalence of mental illness

The Quality and Outcomes Framework (QOF), since its introduction in 2004, has underpinned high quality of recording in primary care. Under the QOF, recording, management and treatment of a large number of clinical domains was financially and reputationally incentivised. The number of domains varied over time with a maximum of 24 clinical domains being included in the scheme for the 2013-14 financial year. We focused on financial year 2015-16, as the most relevant in linking to the latest available primary care payment data (2015-16). Since the introduction of the QOF in 2004, annually updated prevalence data have been available for numerous chronic conditions at the practice level, and these could provide more precise, timely and comprehensive information for determining health care need. (NHS Digital, 2015/16)

## Spatial Weighted analyses and attribution methodology

We used general practice level information from the NHS Digital on several variables of interest to attribute it to our units of analyses, namely the LSOAs. We inputted the LSOA centroid coordinates (longitude and latitude) in R to create a 32,844x32,844 inverse distance matrix (in miles). This matrix features a detailed distance mapping of each LSOA with all other LSOAs and was used to quantify geographical proximity where nearby LSOAs have larger weights and also to generate prevalence for all LSOAs in the period 2015-2016.

In 2014, NHS Digital published for the first time attribution datasets which linked general practice registers to LSOAs and vice versa [[4](#_ENREF_4)]. We used the relevant version of the attribution dataset (i.e. from 2016) to generate annual attribution datasets in 2015-2016. To calculate attribution rates in order to subsequently quantify prevalence and quality of care we used regression modelling under various assumptions to obtain attribution estimates. For each LSOA, if two or more practices were linked to it, we fitted Poisson and negative binomial models with list size and distance to practice as predictors, and the model that was the best fit to the data was selected. If a practice was present both in the analyses and the attribution dataset, we adjusted the attributed population for practice’ list size. If a practice was present in our analyses but not in the attribution dataset, we generated estimates using the models selected in step 1. Redistribution of patients to the other active practices was achieved according to the selected regression model. Finally, the attribution counts estimated in the previous steps across practices were used to generate the weighted mean estimates for prevalence. The algorithm is available from the corresponding author.

## Analysis of antidepressant prescription volume (unadjusted at the regional level and adjusted at the LSOA level)

In the adjusted analysis at the LSOA level, we found large variability between regions for antidepressant prescription volume (Table A3). The North West had 2.38% (95%CI: 2.53% to 2.2%) lower recorded volume of prescribed antidepressants than the North East (reference region). The effect of social fragmentation on antidepressant prescription volume was again opposite than expected, similar to the prevalence of depression analysis. A 1 unit increase in social fragmentation, e.g. from -0.701 (50th centile) to 0.299 (64th centile) was associated with a 1.3% decrease in antidepressant prescription volume within an LSOA. For IMD, a 10 unit increase in the composite measure, e.g. from 17.4 (50th centile) to 27.4 (71th centile) corresponded to a 1.1% increase in antidepressant volume prescription. Urbanity was also a strong predictor, and a very large effect was observed with rural areas associated with 11.4% (95%CI: 12.3% to 10.5%) lower antidepressant prescription volume. The highest prevalence of SMI was observed for the 65plus age group.

## Sensitivity analyses with interaction terms

In an additional sensitivity analyses we examined whether the associations between outcomes and deprivation and social fragmentation varied across regions. For depression we found evidence of small variability in the association between regions and social fragmentation. More specifically, the interaction terms between social fragmentation and region varied from -0.004 (Yorkshire and Humber) to 0.153 (South East). Even though the effects were small, we found evidence that the effects of fragmentation on prevalence of depression vary across regions. For example this indicates that more fragmented areas in the South East will require more attention when compared to socially fragmented areas in Yorkshire and Humber and a potential reorganisation of care should take this into account. Areas such as the South East, London or East England appear to be areas where social fragmentation has the largest effect on the prevalence of depression. For the interaction between the IMD and regions, variability was very small and varied from 0.005 (South Central) to 0.032 (South West). The very small effects of interactions between the IMD and regions may also provide more evidence that social fragmentation is more important than deprivation when explaining variations in the prevalence of depression. Finally, for SMI we observed very little to no variability in the associations between social fragmentation, deprivation and regions.

Table A1: Results from model B for depression, linear regression at the LSOA level, with region-social fragmentation and region-IMD interactions*†‡

|  | coefficient | 95% Confidence Interval | | p-value |
| --- | --- | --- | --- | --- |
| Region | | | | |
| North East | reference | | | |
| North West | -0.052 | -0.264 | 0.159 | 0.627 |
| Yorkshire & Humber | -0.558 | -0.775 | -0.341 | 0.001 |
| East Midlands | 0.223 | -0.003 | 0.451 | 0.054 |
| West Midlands | -0.425 | -0.647 | -0.203 | 0.001 |
| East England | -0.915 | -1.135 | -0.694 | 0.001 |
| London | -2.462 | -2.681 | -2.243 | 0.001 |
| South East | -0.592 | -0.816 | -0.368 | 0.001 |
| South Central | -0.344 | -0.567 | -0.120 | 0.001 |
| South West | -0.954 | -1.174 | -0.733 | 0.001 |
| Demographics† | | | | |
| % aged 25-44, 2015 | 0.029 | 0.024 | 0.034 | 0.001 |
| % aged 45-64, 2015 | 0.017 | 0.010 | 0.023 | 0.001 |
| % aged 65 or over, 2015 | -0.017 | -0.022 | -0.013 | 0.001 |
| % Female, 2015 | 0.004 | -0.004 | 0.014 | 0.315 |
| % White British, 2011 | 0.045 | 0.044 | 0.047 | 0.001 |
| LSOA Metrics† | | | | |
| Rural LSOA | -0.780 | -0.842 | -0.718 | 0.001 |
| Social Fragmentation 2011 | -0.138 | -0.168 | -0.108 | 0.001 |
| IMD 2015 | 0.021 | 0.015 | 0.027 | 0.001 |
| constant | 3.249 | 2.662 | 3.835 | 0.001 |
| Region#Social Fragmentation interaction‡ | | | | |
| North East | Reference | | | |
| North West | 0.057 | 0.022 | 0.092 | 0.001 |
| Yorkshire & Humber | -0.004 | -0.038 | 0.030 | 0.821 |
| East Midlands | 0.012 | -0.024 | 0.049 | 0.508 |
| West Midlands | 0.052 | 0.015 | 0.089 | 0.005 |
| East England | 0.084 | 0.045 | 0.122 | 0.001 |
| London | 0.091 | 0.056 | 0.125 | 0.001 |
| South East | 0.153 | 0.115 | 0.191 | 0.001 |
| South Central | 0.024 | -0.013 | 0.062 | 0.215 |
| South West | 0.078 | 0.042 | 0.115 | 0.001 |
| Region#IMD interaction‡ | | | | |
| North East | Reference | | | |
| North West | 0.026 | 0.019 | 0.033 | 0.001 |
| Yorkshire & Humber | 0.018 | 0.010 | 0.025 | 0.001 |
| East Midlands | 0.009 | 0.001 | 0.017 | 0.015 |
| West Midlands | 0.015 | 0.008 | 0.023 | 0.001 |
| East England | 0.013 | 0.005 | 0.021 | 0.001 |
| London | 0.028 | 0.020 | 0.035 | 0.001 |
| South East | 0.024 | 0.015 | 0.033 | 0.001 |
| South Central | 0.005 | -0.004 | 0.014 | 0.283 |
| South West | 0.032 | 0.024 | 0.041 | 0.001 |

** 32844 LSOAs (observations) with analytic weighting, adjusted R-squared=34.22%*

*† Coefficients reported are for the reference region only, North East*

*‡ Differences to the coefficients reported for the reference region*

Table A2: Results from model B for SMI, linear regression at the LSOA level, with region-social fragmentation and region-IMD interactions*†‡

|  | coefficient | 95% Confidence Interval | | p-value |
| --- | --- | --- | --- | --- |
| Region | | | | |
| North East | Reference | | | |
| North West | -0.003 | -0.025 | 0.018 | 0.748 |
| Yorkshire & Humber | -0.098 | -0.121 | -0.076 | 0.001 |
| East Midlands | -0.131 | -0.155 | -0.108 | 0.001 |
| West Midlands | -0.152 | -0.175 | -0.129 | 0.001 |
| East England | -0.097 | -0.120 | -0.074 | 0.001 |
| London | -0.143 | -0.166 | -0.120 | 0.001 |
| South East | -0.084 | -0.107 | -0.060 | 0.001 |
| South Central | -0.138 | -0.162 | -0.115 | 0.001 |
| South West | -0.082 | -0.105 | -0.059 | 0.001 |
| Demographics† | | | | |
| % aged 25-44, 2015 | 0.005 | 0.004 | 0.005 | 0.001 |
| % aged 45-64, 2015 | 0.011 | 0.010 | 0.011 | 0.001 |
| % aged 65 or over, 2015 | 0.005 | 0.005 | 0.006 | 0.001 |
| % Female, 2015 | 0.002 | 0.001 | 0.003 | 0.001 |
| % White British, 2011 | -0.003 | -0.003 | -0.003 | 0.001 |
| LSOA Metrics† | | | | |
| Rural LSOA | -0.122 | -0.128 | -0.116 | 0.001 |
| Social Fragmentation 2011 | 0.011 | 0.008 | 0.015 | 0.001 |
| IMD 2015 | 0.003 | 0.002 | 0.004 | 0.001 |
| constant | 0.498 | 0.436 | 0.559 | 0.001 |
| Region#Social Fragmentation interaction‡ | | | | |
| North East | Reference | | | |
| North West | 0.006 | 0.003 | 0.010 | 0.001 |
| Yorkshire & Humber | 0.000 | -0.003 | 0.003 | 0.865 |
| East Midlands | 0.002 | -0.001 | 0.006 | 0.282 |
| West Midlands | 0.002 | -0.001 | 0.006 | 0.150 |
| East England | 0.009 | 0.005 | 0.013 | 0.001 |
| London | 0.024 | 0.020 | 0.028 | 0.001 |
| South East | 0.020 | 0.016 | 0.024 | 0.001 |
| South Central | 0.010 | 0.006 | 0.014 | 0.001 |
| South West | 0.014 | 0.010 | 0.018 | 0.001 |
| Region#IMD interaction‡ | | | | |
| North East | Reference | | | |
| North West | 0.002 | 0.001 | 0.003 | 0.001 |
| Yorkshire & Humber | 0.001 | 0.000 | 0.001 | 0.003 |
| East Midlands | 0.002 | 0.001 | 0.003 | 0.001 |
| West Midlands | 0.003 | 0.002 | 0.004 | 0.001 |
| East England | 0.002 | 0.001 | 0.003 | 0.001 |
| London | 0.006 | 0.005 | 0.006 | 0.001 |
| South East | 0.002 | 0.001 | 0.003 | 0.001 |
| South Central | 0.003 | 0.002 | 0.004 | 0.001 |
| South West | 0.002 | 0.001 | 0.003 | 0.001 |

** 32844 LSOAs (observations) with analytic weighting, adjusted R-squared=46.44%*

*† Coefficients reported are for the reference region only, North East*

*‡ Differences to the coefficients reported for the reference region*

| Table A3: Results from model A for Antidepressant Volume Prescription Comparators (Antidepressant ADQ/STARPU), linear regression at the LSOA level*†‡ | | | | |
| --- | --- | --- | --- | --- |
| **Outcome: Prevalence of SMI** | **coefficient** | **95% Confidence Interval** | | **p-value** |
| *Region* | | | | |
| *North East* | *Reference* | | | |
| *North West* | -0.238 | -0.253 | -0.222 | 0.001 |
| *Yorkshire & Humber* | -0.473 | -0.490 | -0.457 | 0.001 |
| *East Midlands* | -0.536 | -0.553 | -0.519 | 0.001 |
| *West Midlands* | -0.664 | -0.680 | -0.647 | 0.001 |
| *East-England* | -0.594 | -0.610 | -0.577 | 0.001 |
| *London* | -0.962 | -0.979 | -0.944 | 0.001 |
| *South East* | -0.693 | -0.710 | -0.675 | 0.001 |
| *South Central* | -0.568 | -0.586 | -0.551 | 0.001 |
| *South West* | -0.592 | -0.608 | -0.575 | 0.001 |
| Demographics | | | | |
| *% aged 25-44, 2015* | 0.0014 | 0.0007 | 0.0020 | 0.001 |
| *% aged 45-64, 2015* | -0.0018 | -0.0028 | -0.0009 | 0.001 |
| *% aged 65 plus, 2015* | 0.0032 | 0.002 | 0.003 | 0.001 |
| *% female, 2015* | -0.006 | -0.007 | -0.004 | 0.001 |
| *% white British, 2015* | 0.011 | 0.010 | 0.011 | 0.001 |
| LSOA Metrics | | | | |
| *Rural LSOA* | -0.114 | -0.122 | -0.104 | 0.001 |
| *Social Fragmentation* | -0.013 | -0.015 | -0.012 | 0.001 |
| *IMD 2015* | 0.011 | 0.011 | 0.011 | 0.001 |
| *Constant* | 1.198 | 1.118 | 1.279 | 0.001 |

** 32844 LSOAs (observations) with analytic weighting*

*† adjusted R-squared=68.26%*

*‡ coefficients can be interpreted as percentage change, for example adjusted antidepressant volume levels in the North West were 0.238% lower than in the North East*

Figure 1A: Box plots of prevalence of Depression for 2015-16 [top] and prevalence of Severe Mental Illness for 2015-16 [bottom], across English regions* [weighted for LSOA size 2015].
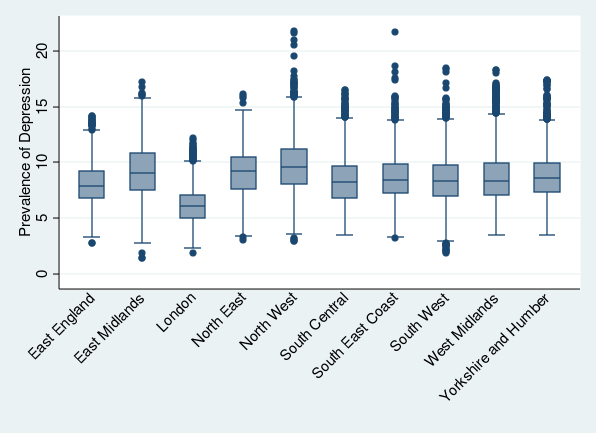

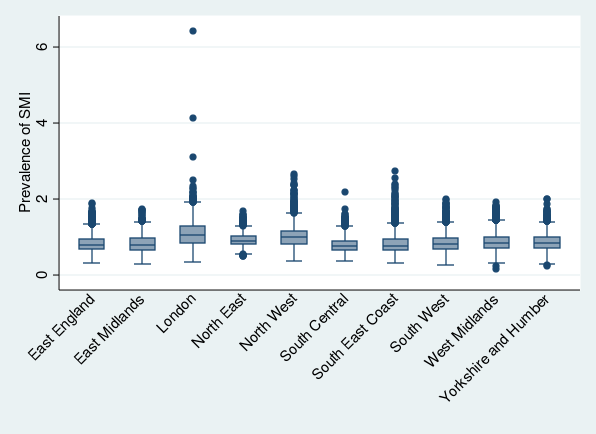


Figure 2A: Spatial clustering [Moran’s I with 95% CIs] for 2015-16 Prevalence of Depression, 2015-2016 Prevalence of SMI, and the 2011 Index of Social Fragmentation, within each region and for the whole of England.


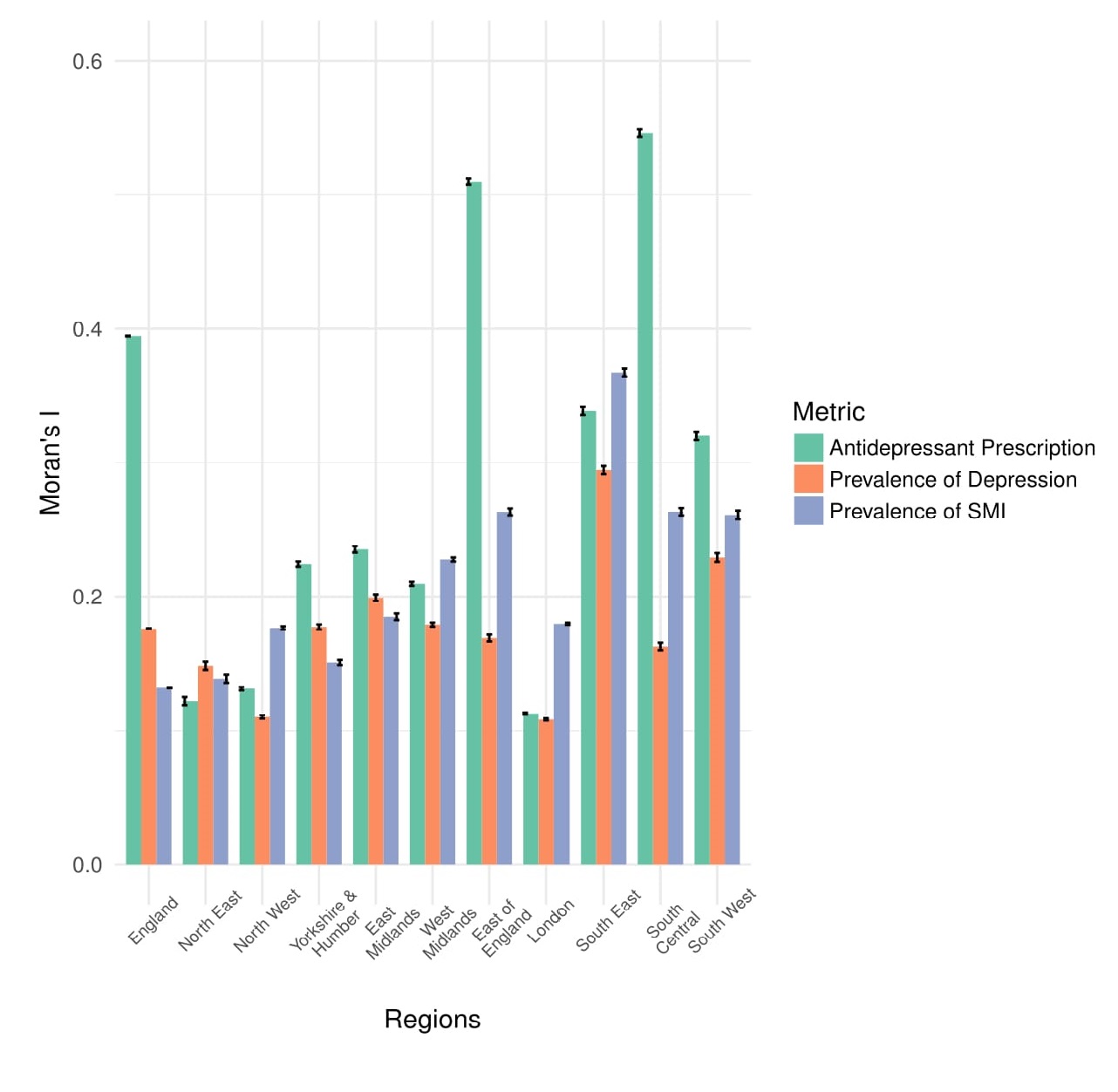


Figure 3A: Antidepressant prescription volume in England (2015/16 LSOA level).


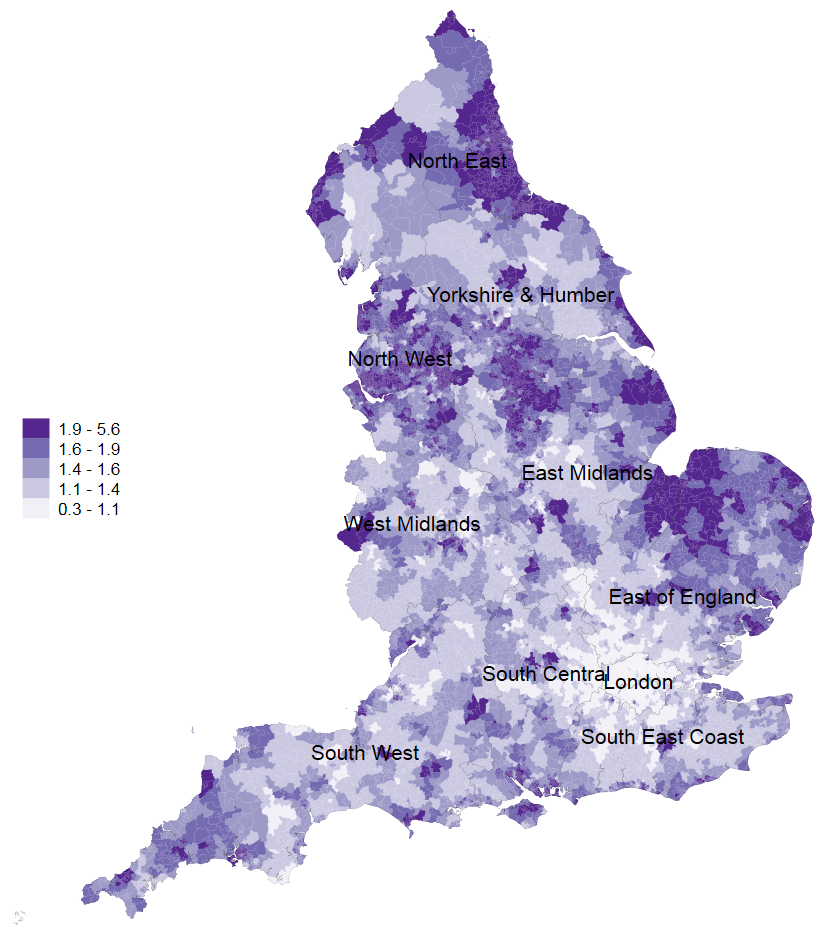


Figure A1: East England

##
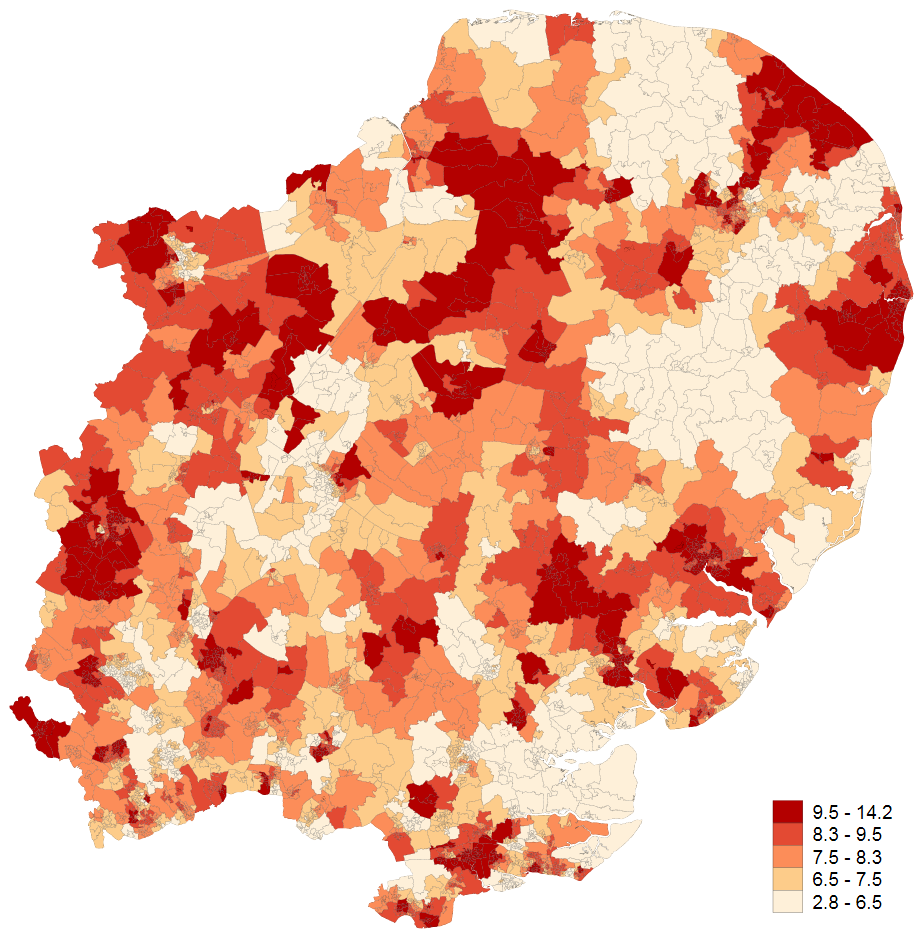


Figure A1: East England

##
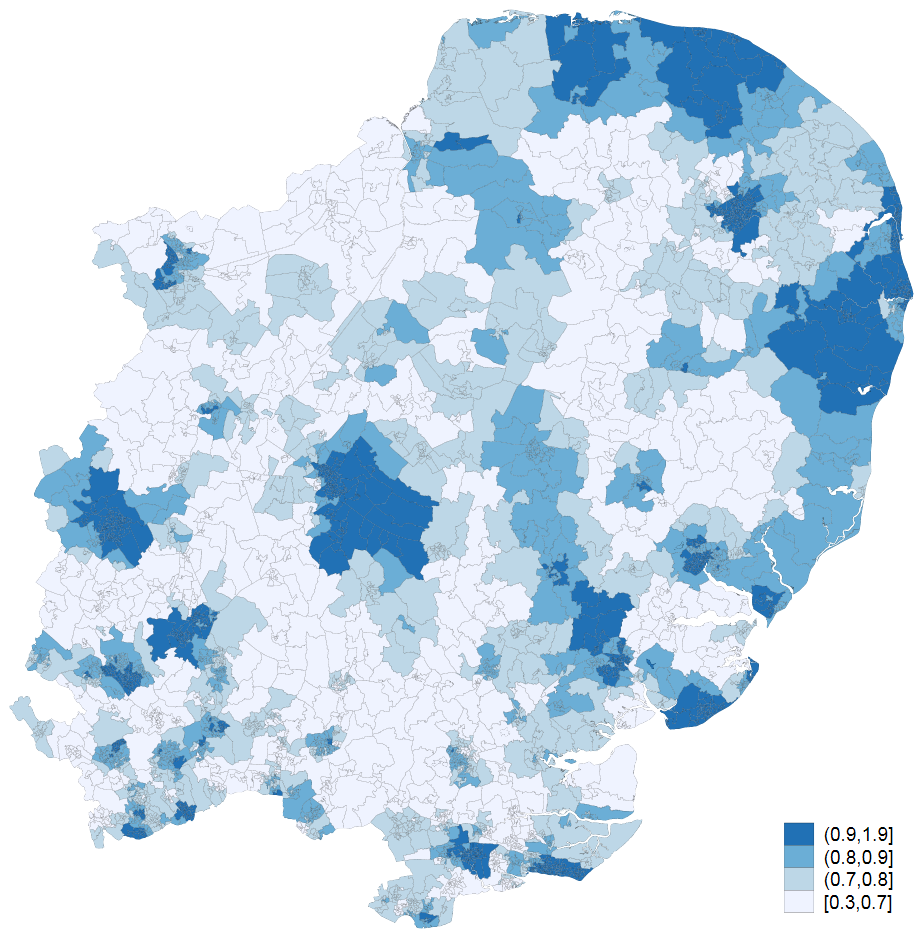


Figure B1: East Midlands


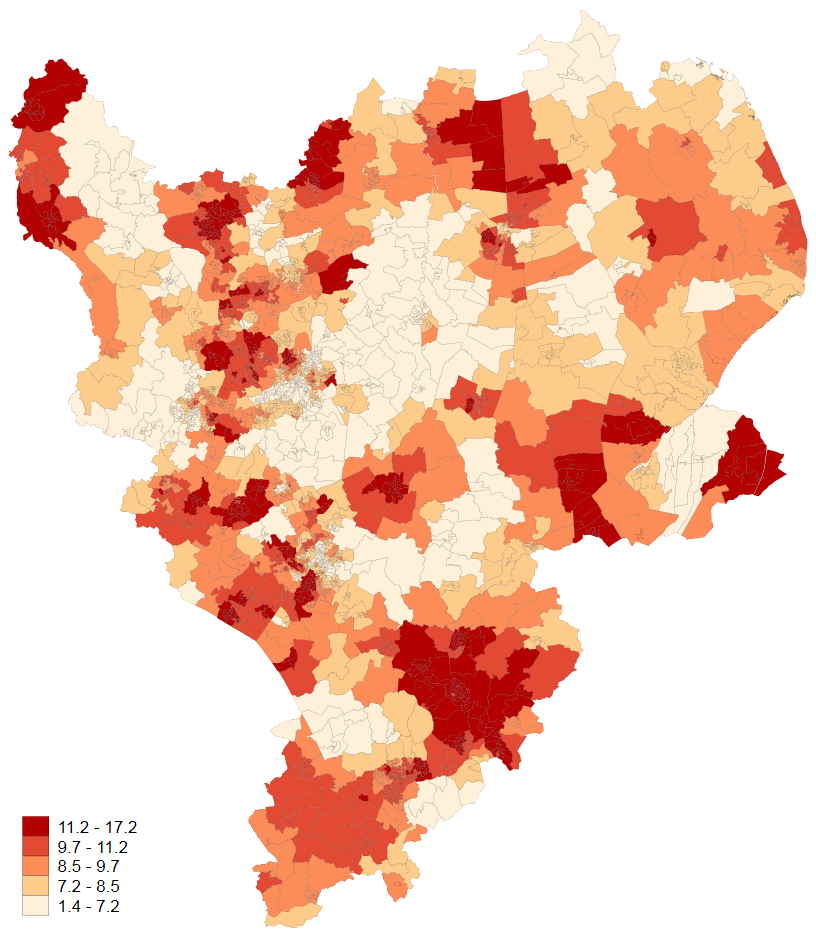


Figure B2: East Midlands

**
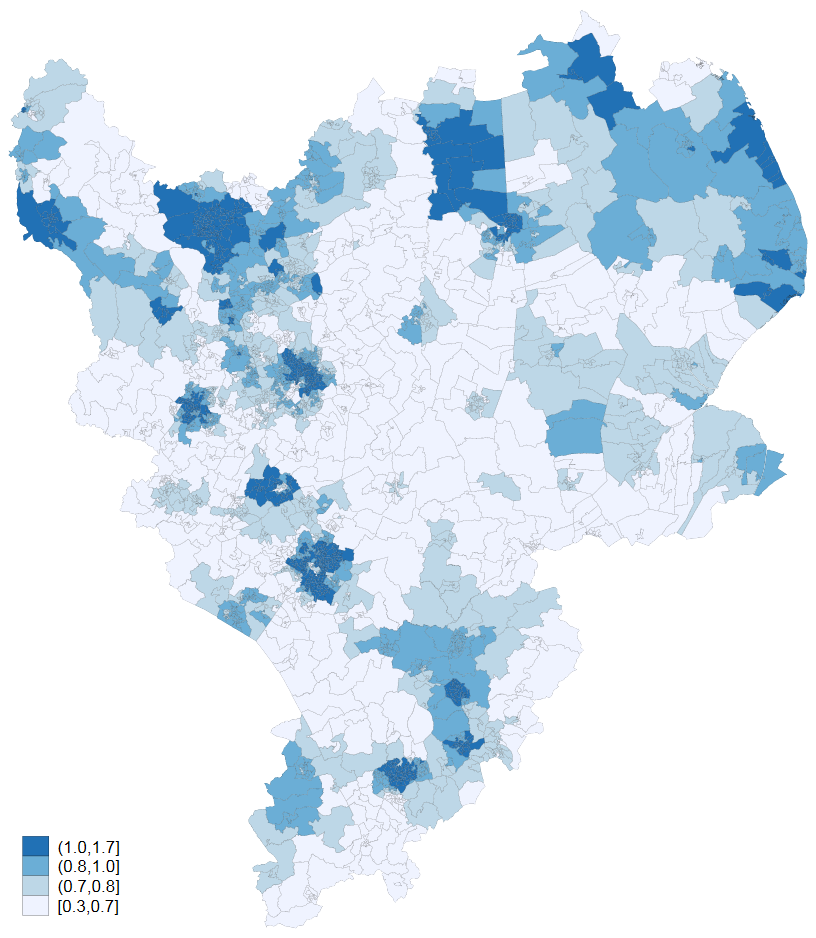
**

Figure C1: North East


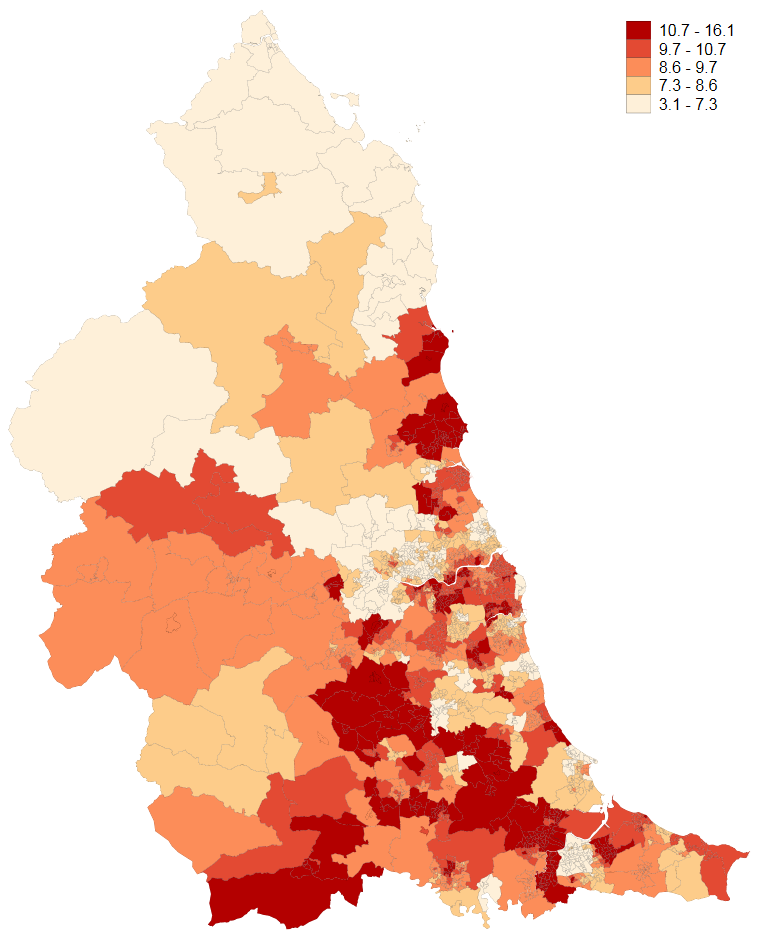


Figure C2: North East


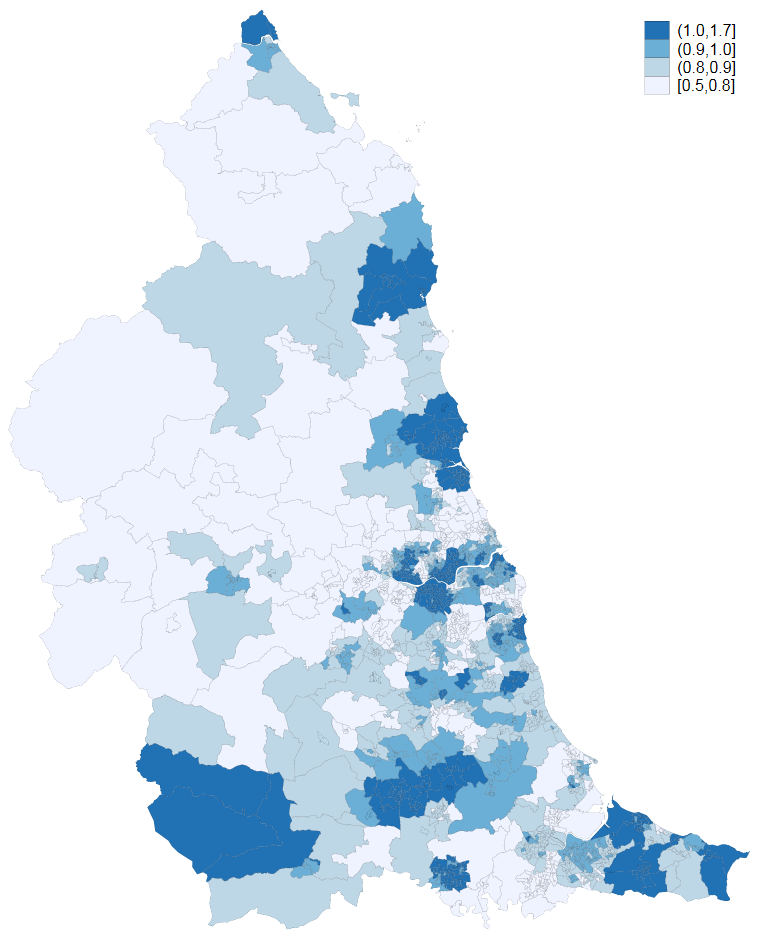


Figure D1: North West

##
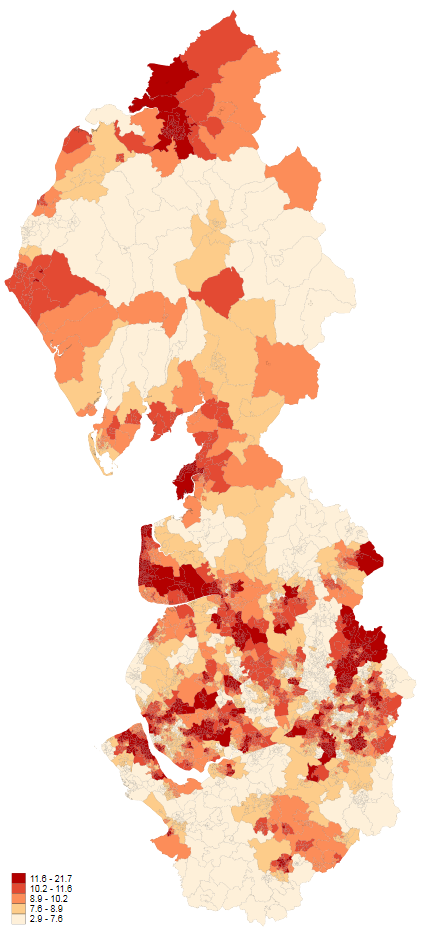


## Figure D2: North West

##
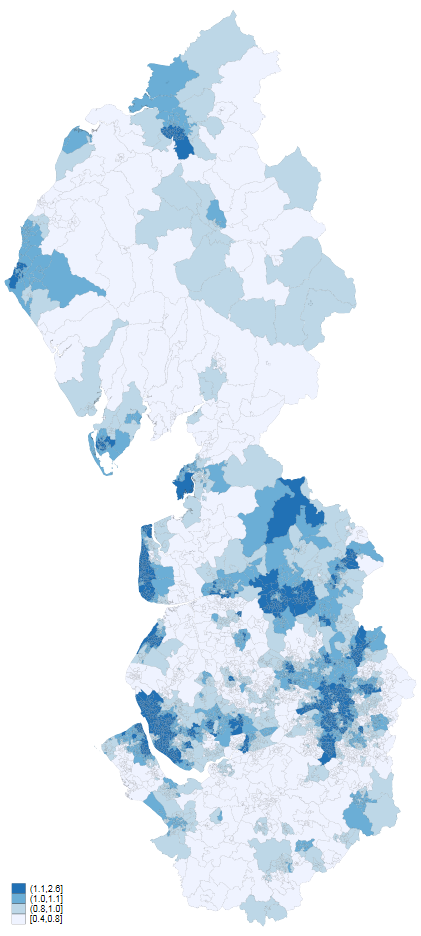


Figure E1: South East Coast


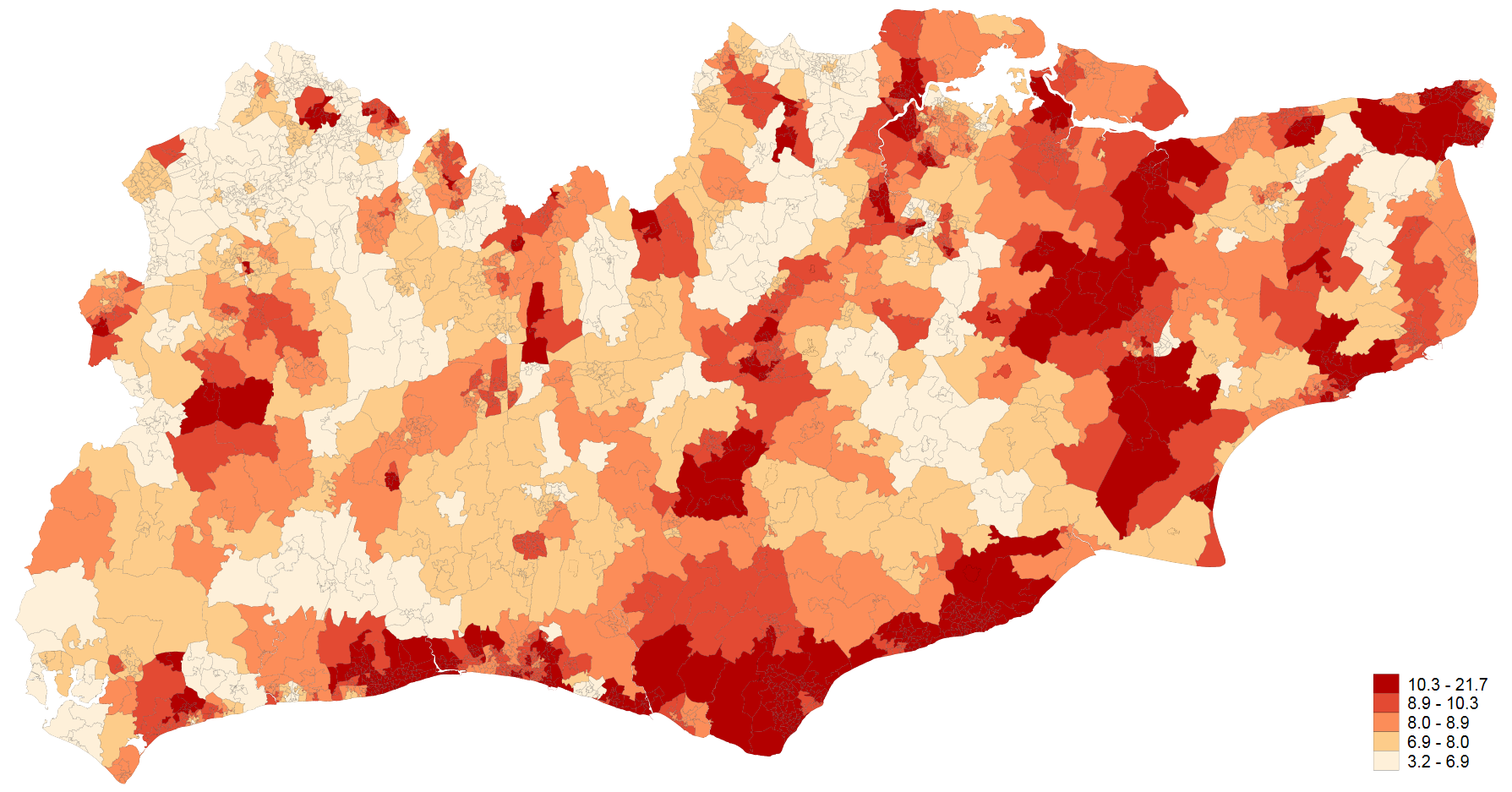


Figure E2: South East Coast


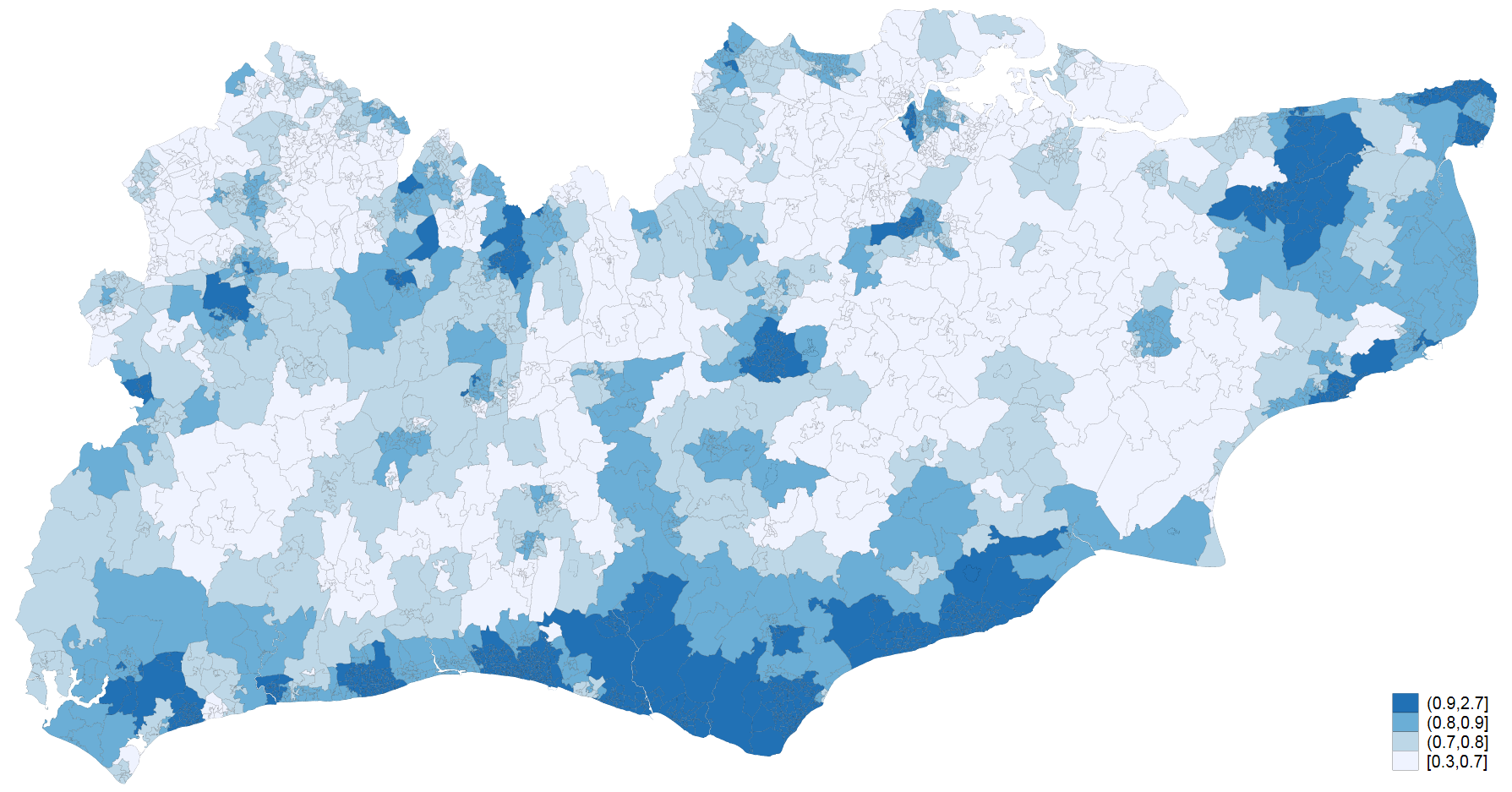


Figure F1: South Central
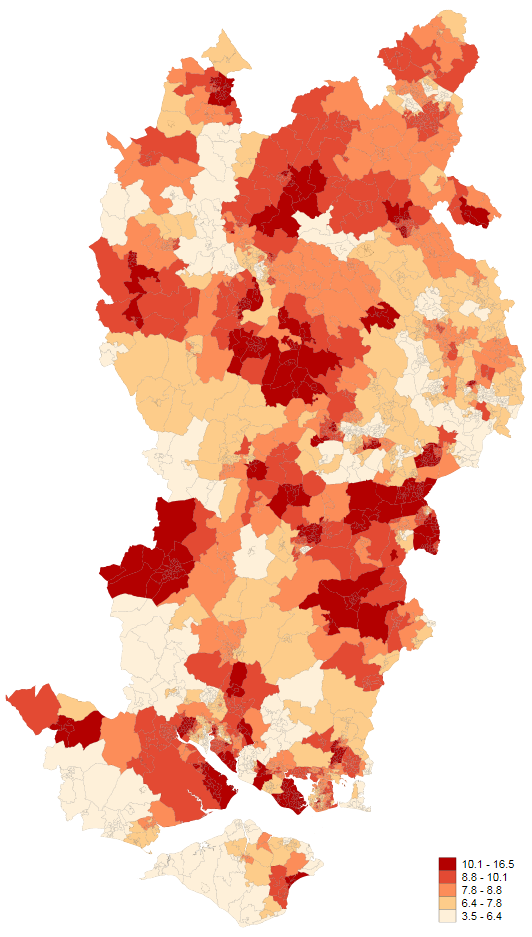


## Figure F2: South Central


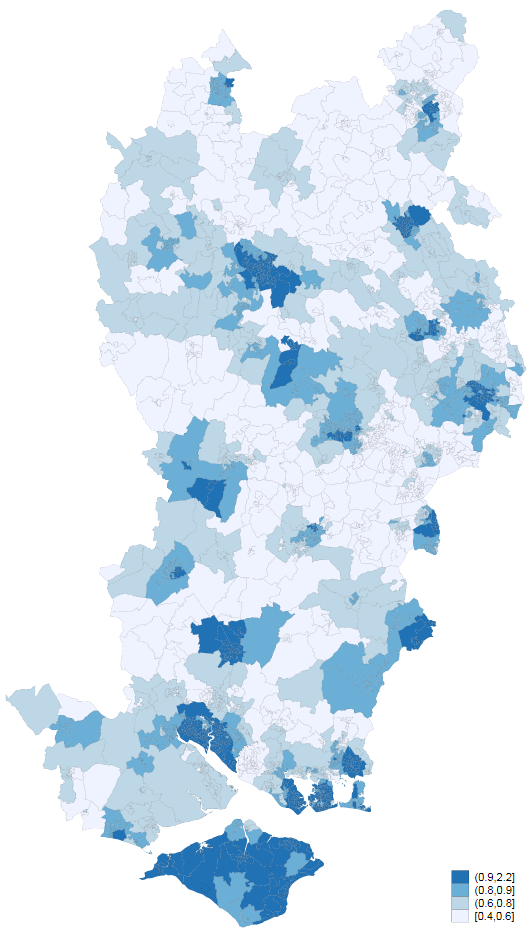


**Figure G1: Yorkshire & Humber**


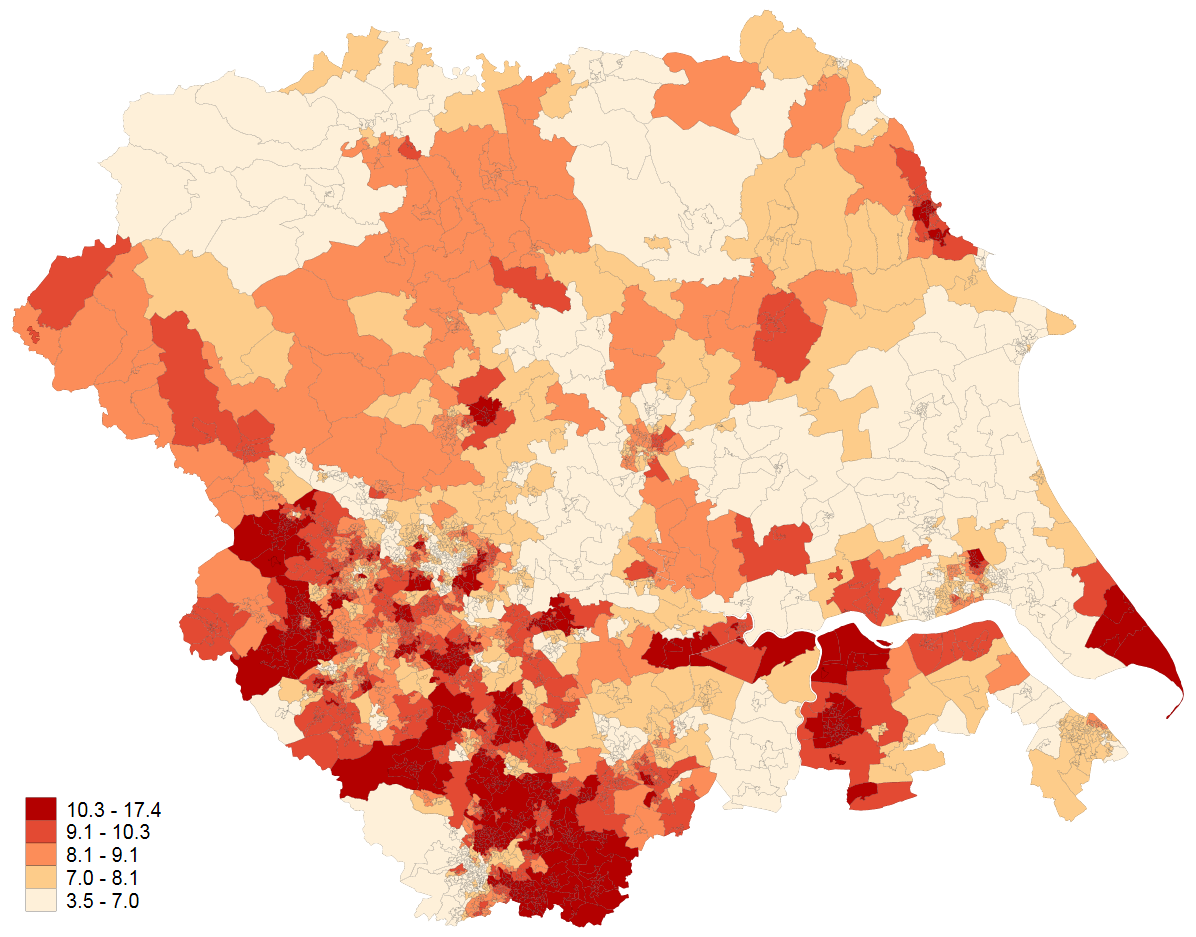


**Figure G2: Yorkshire & Humber**

**
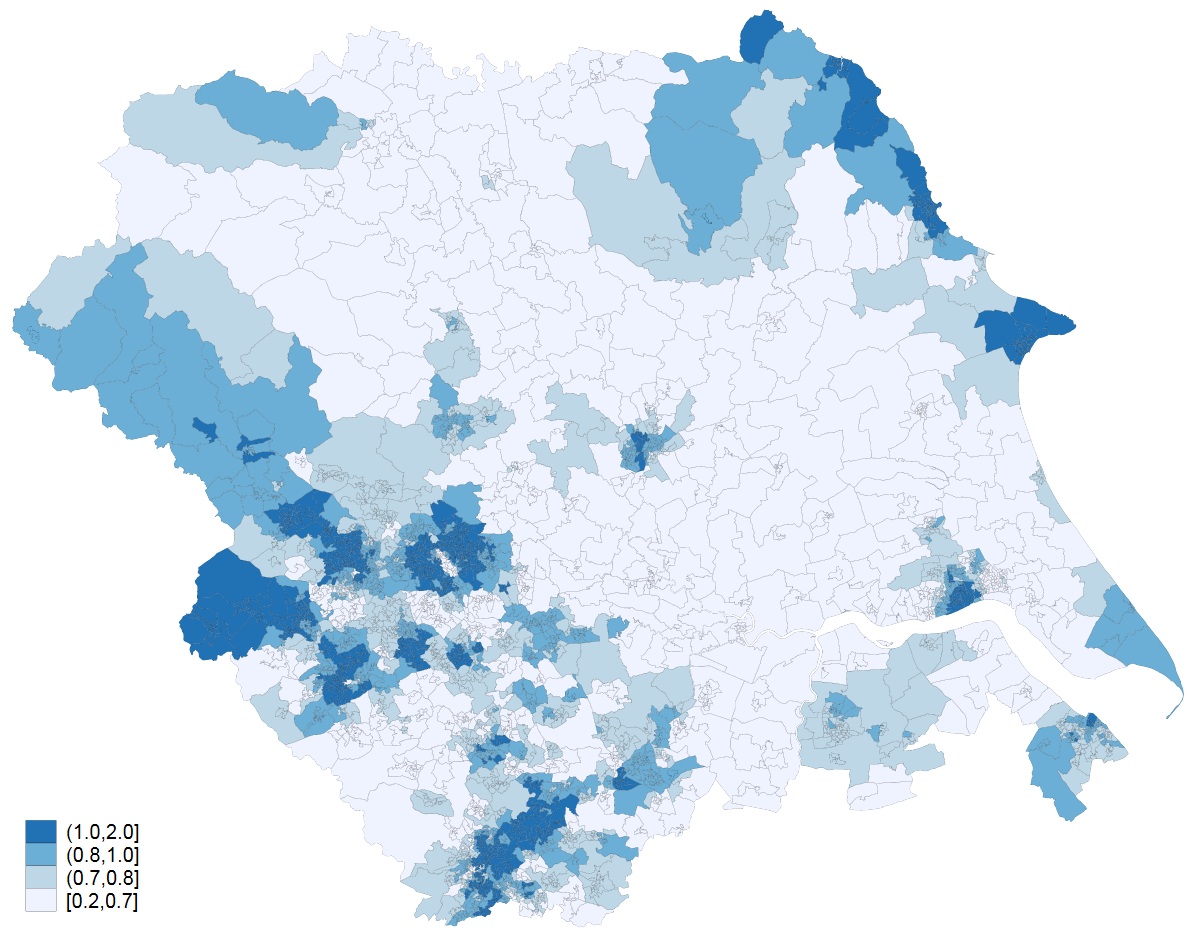
**

**Figure H1: West Midlands**

**
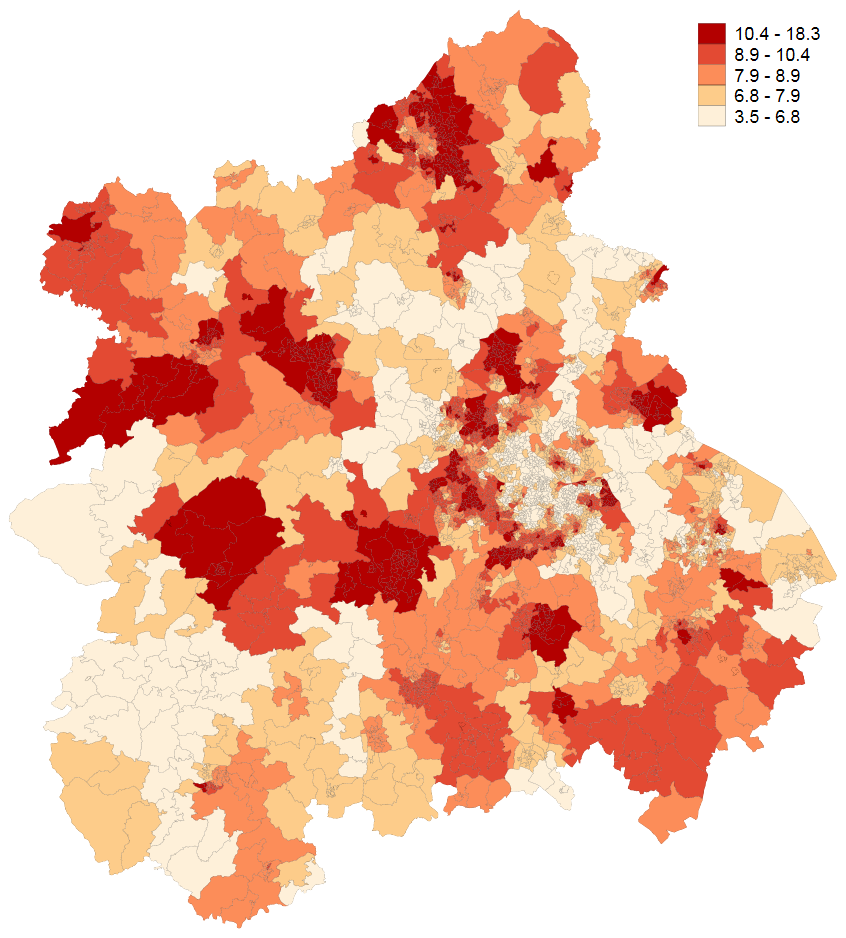
**

**Figure H2: West Midlands**

**
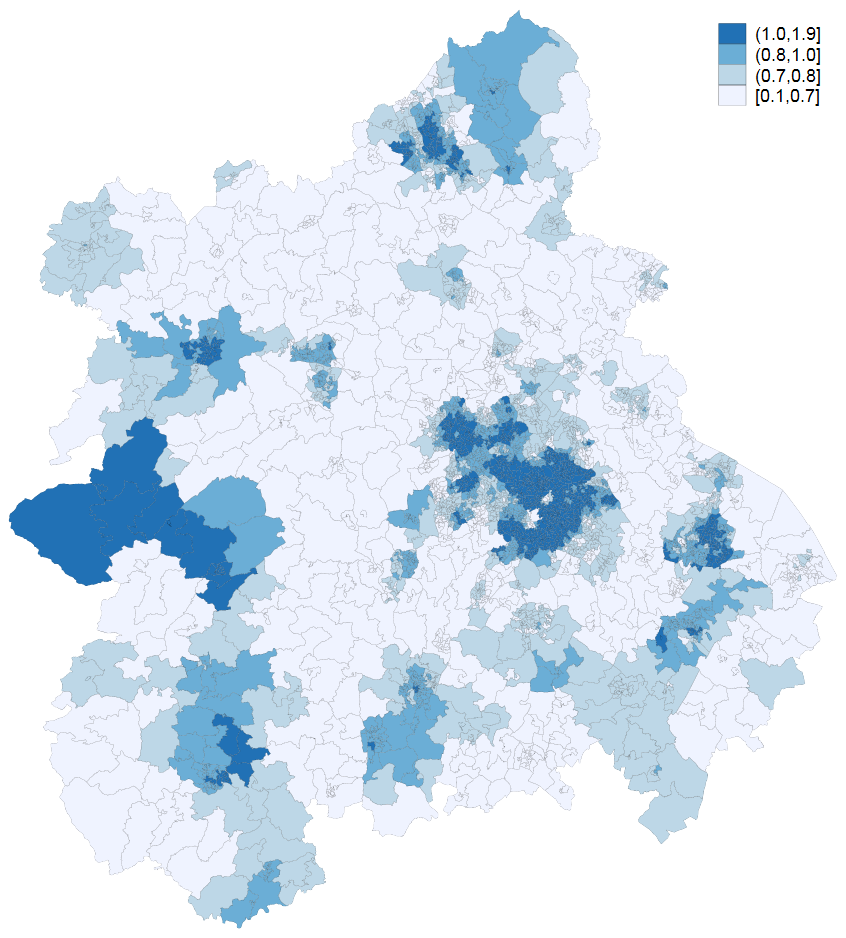
**

**Figure I1: London**

**
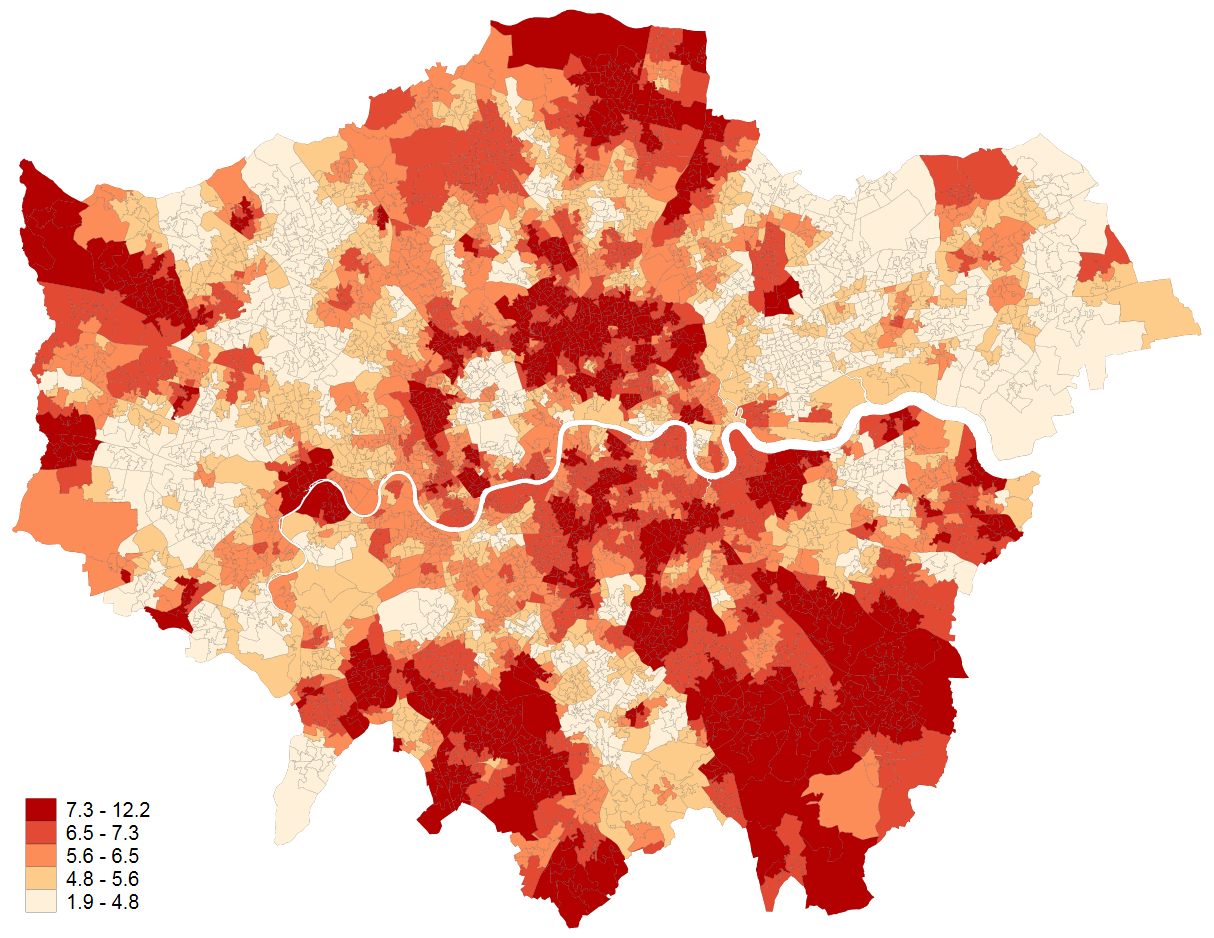
**

**Figure I2: London**


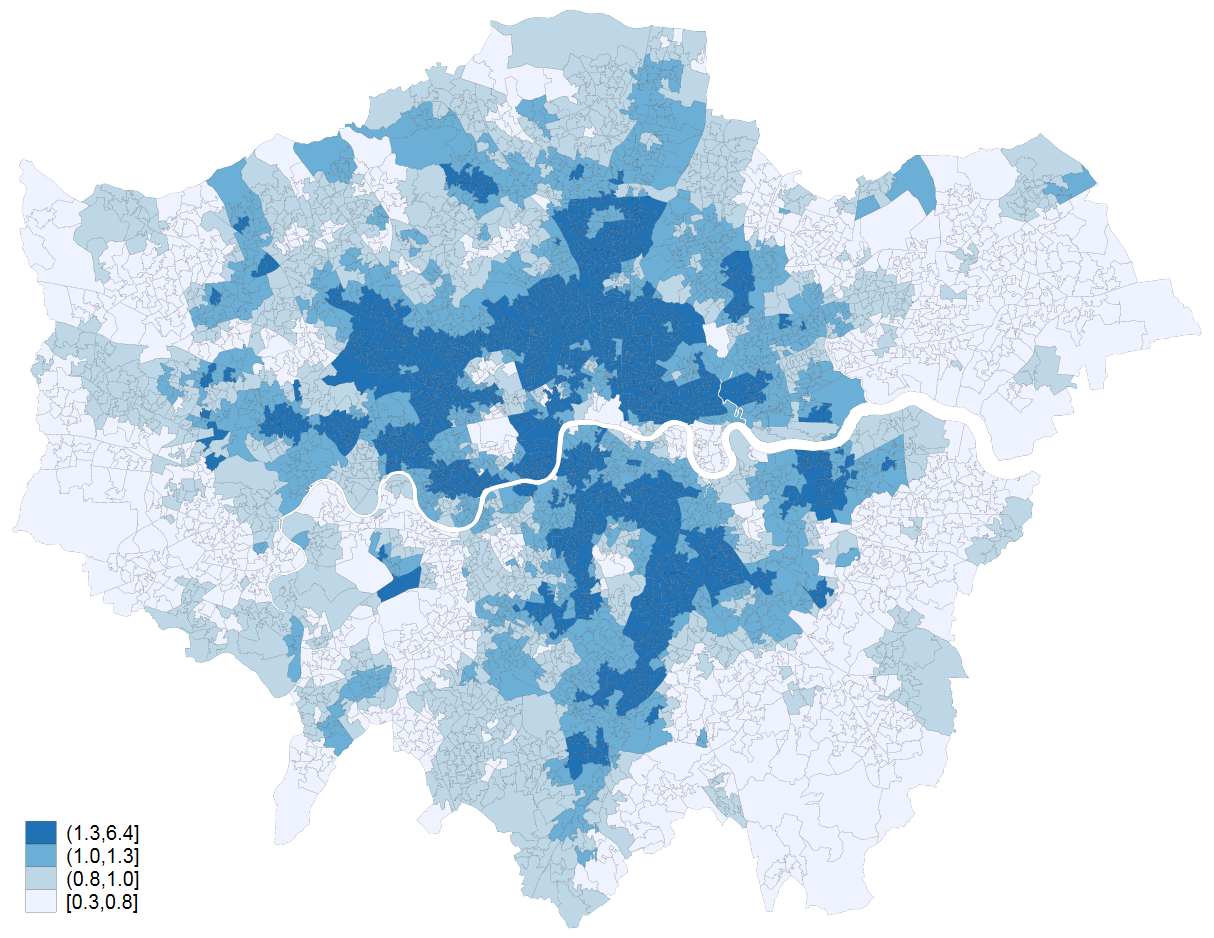


**Figure J1: South West**


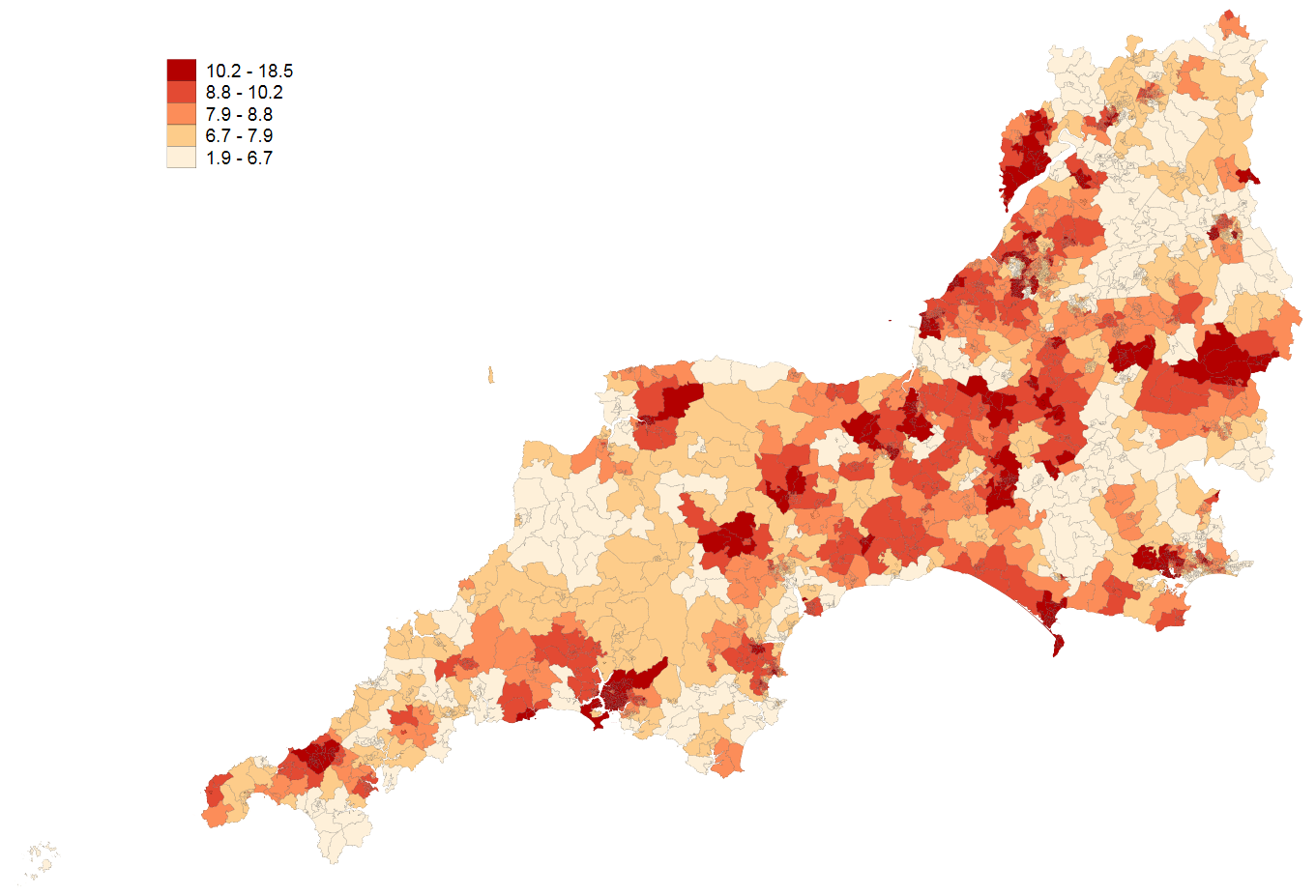


**Figure J2: South West**


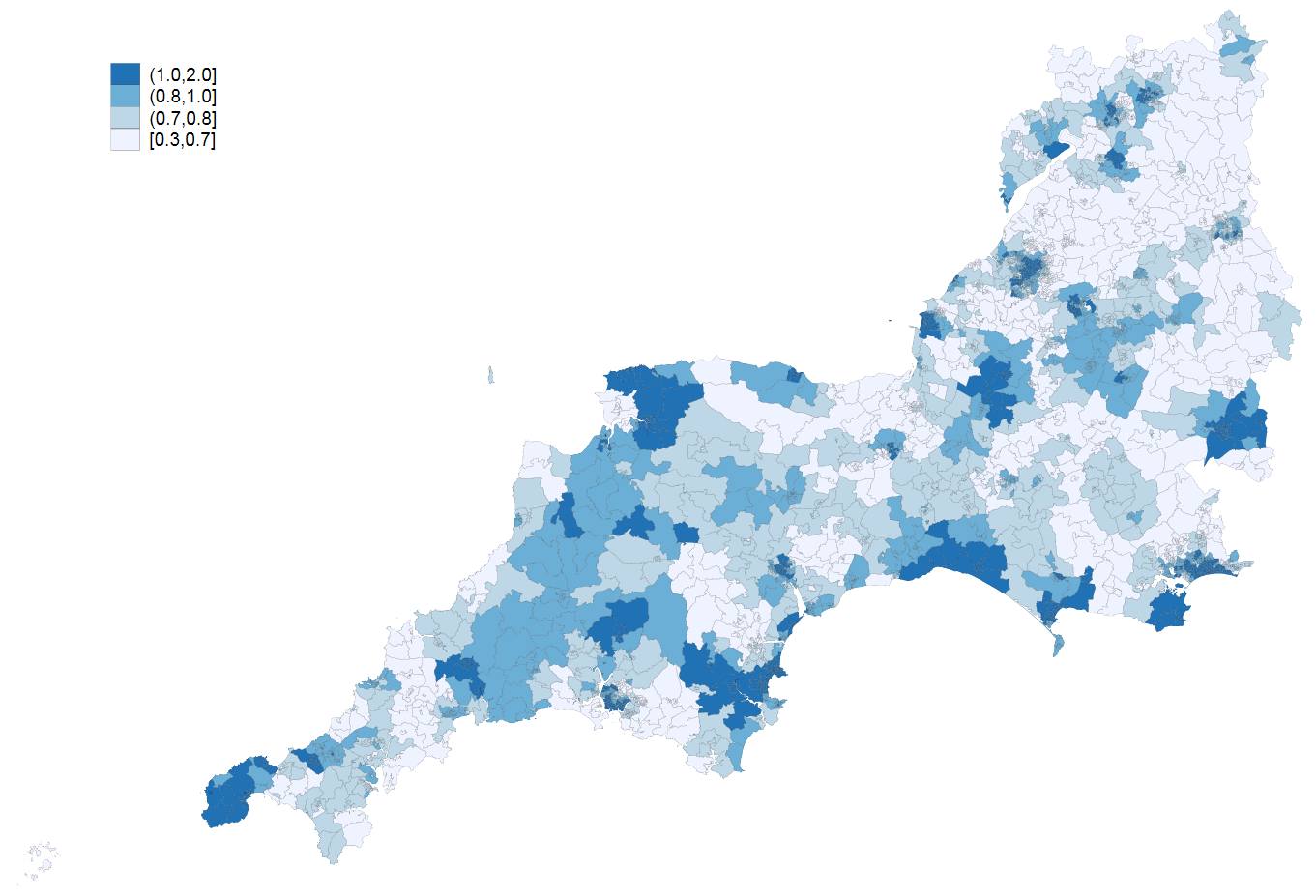


Figure A3: East England


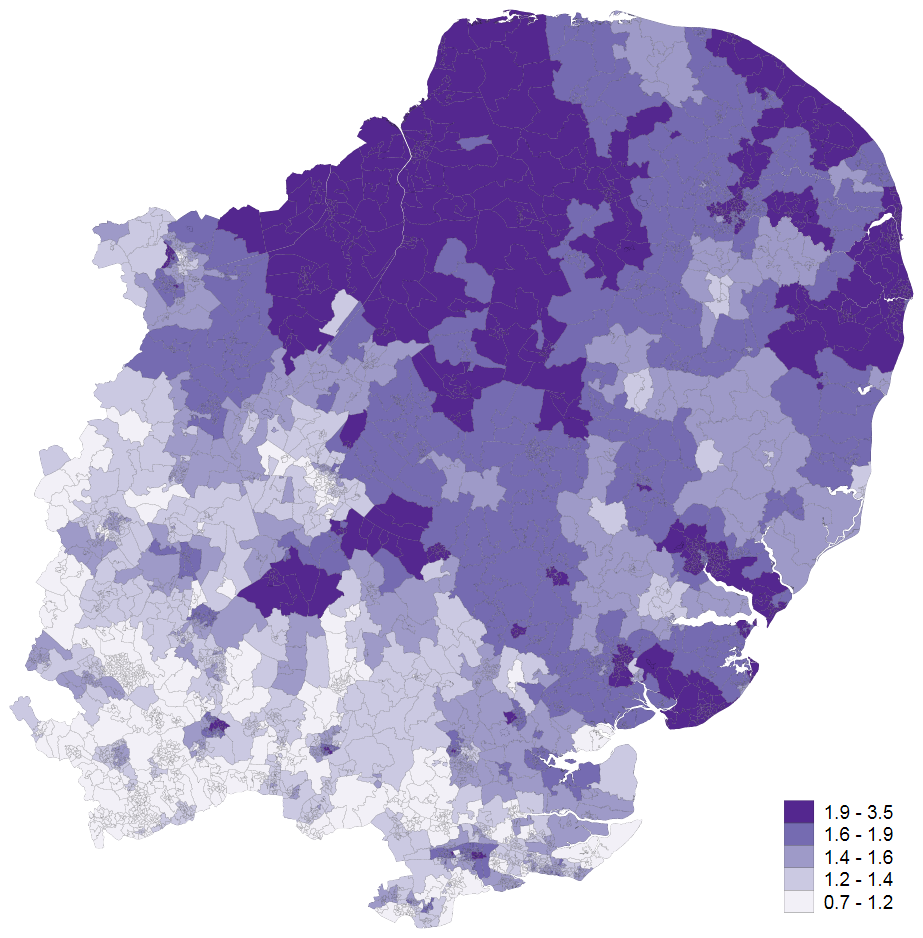


Figure B3: East Midlands
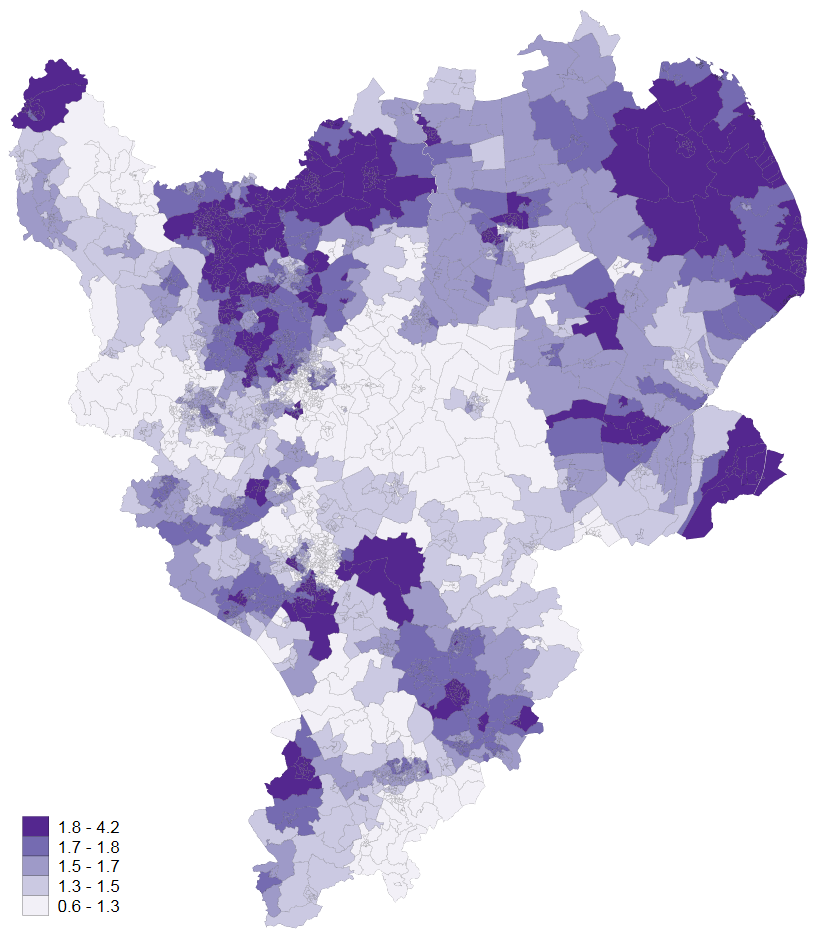


Figure C3: North East


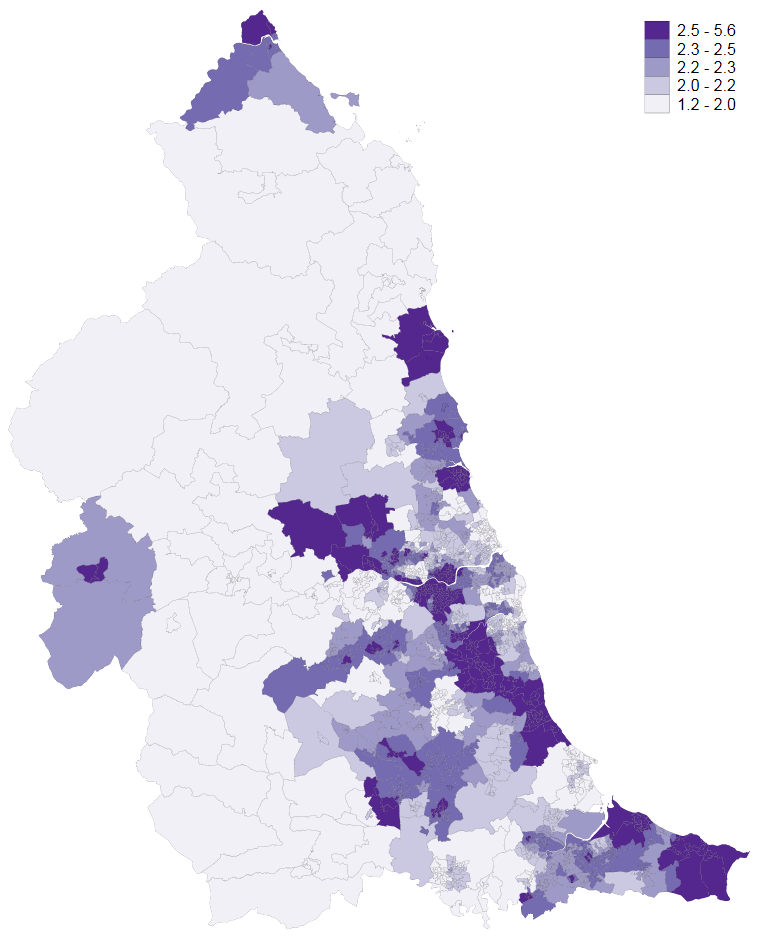


Figure D3: North West


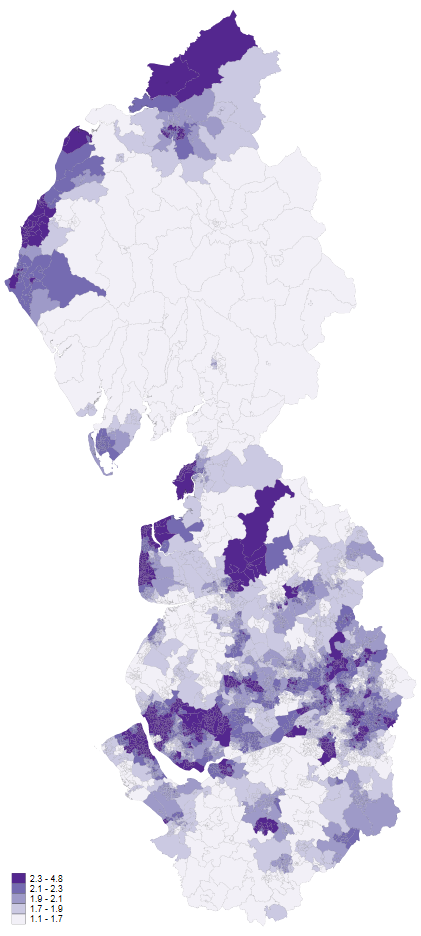


Figure E3: South East Coast


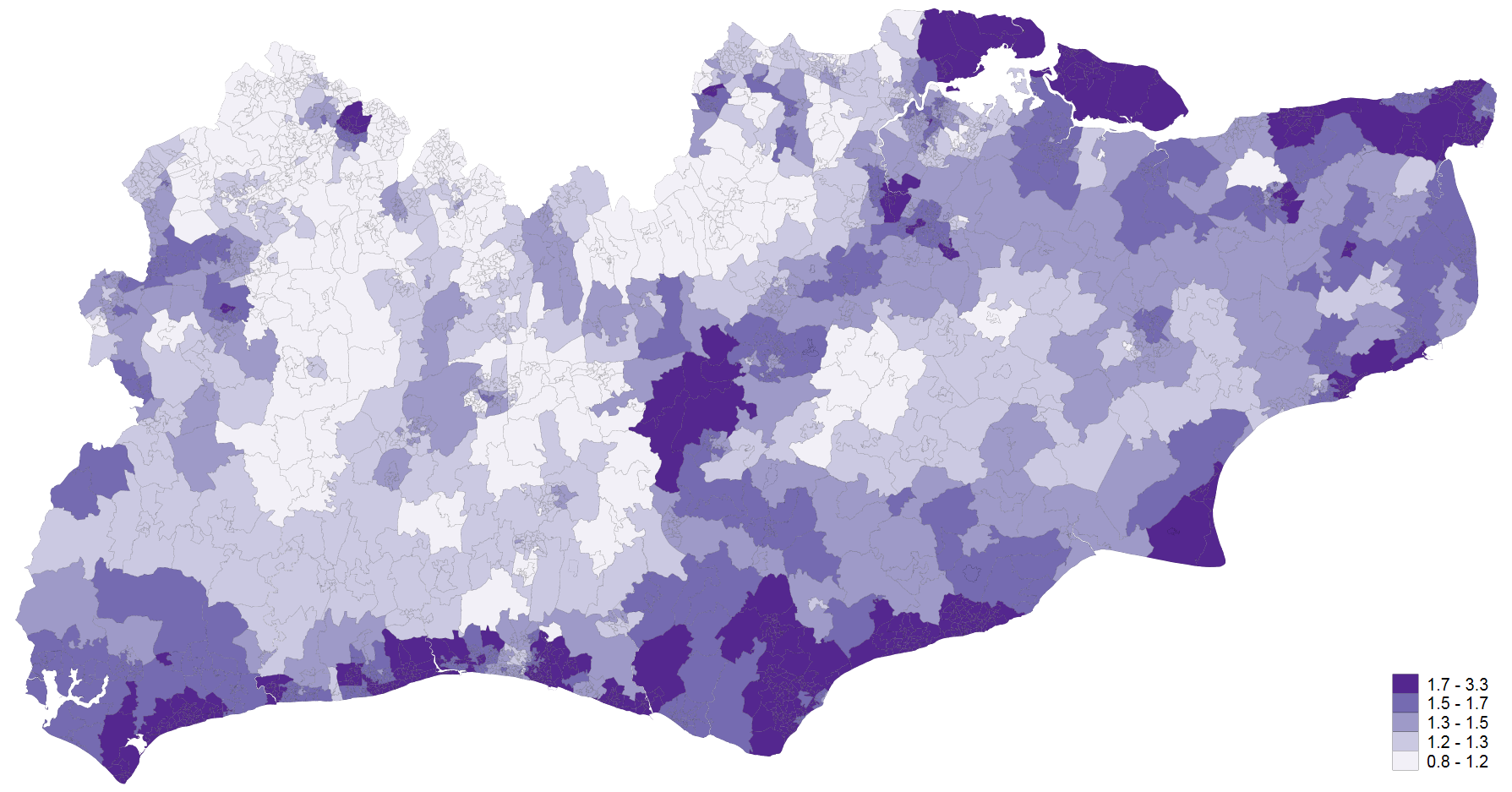


## Figure F3: South Central

##
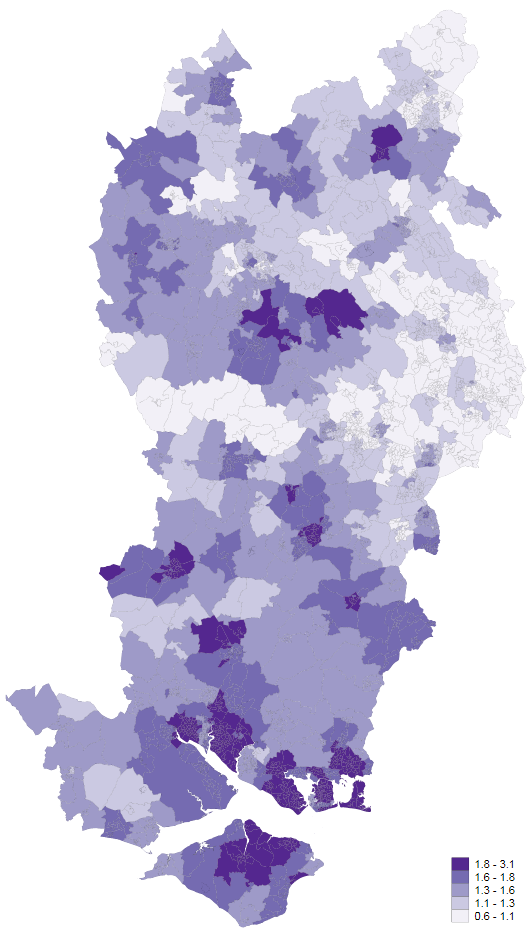


**Figure G3: Yorkshire & Humber**

**
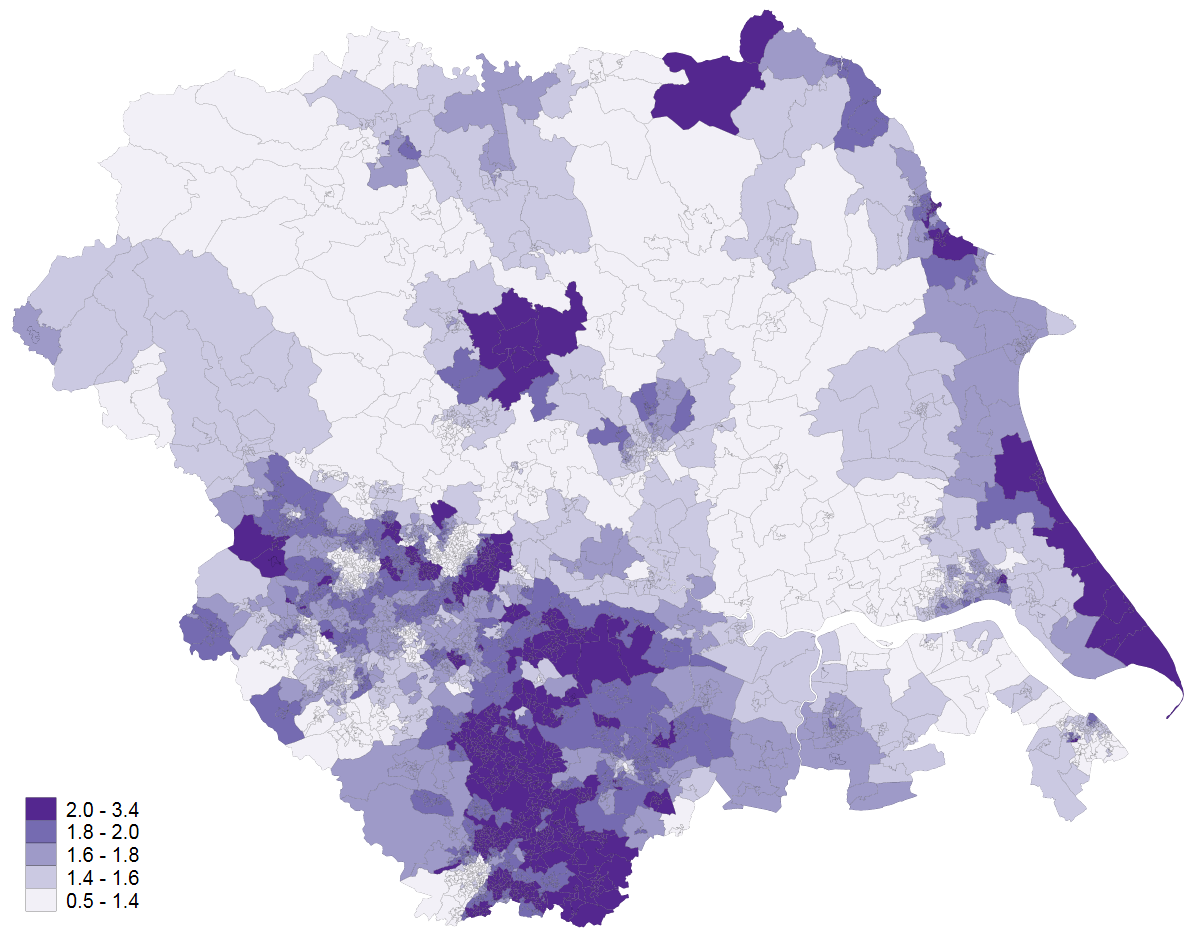
**

**Figure H3: West Midlands
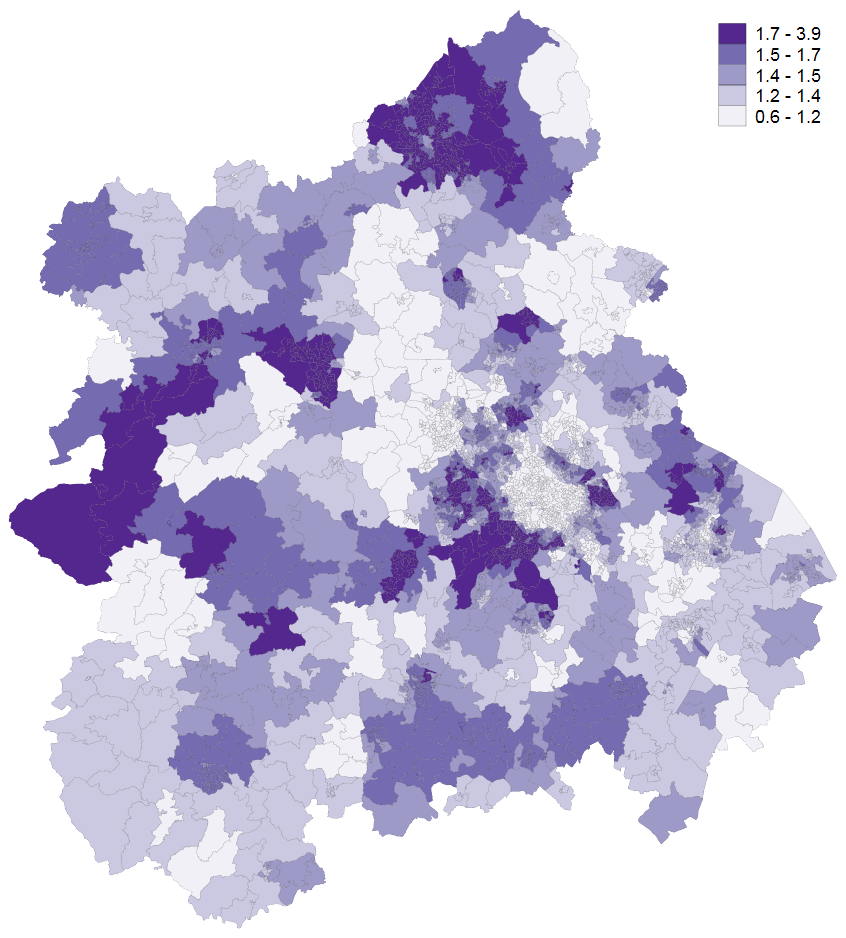
**

**Figure I3: London**


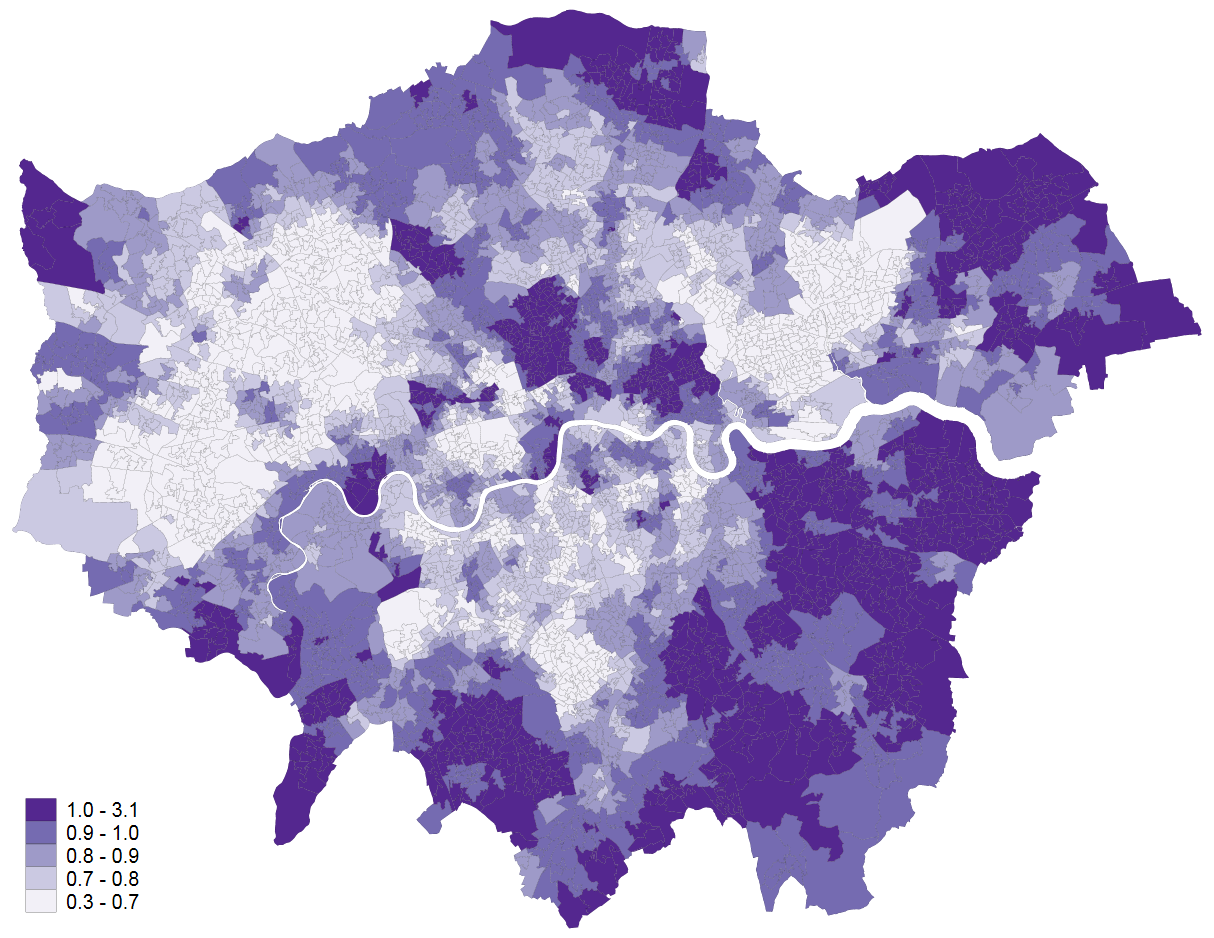


**Figure J3: South West**

**
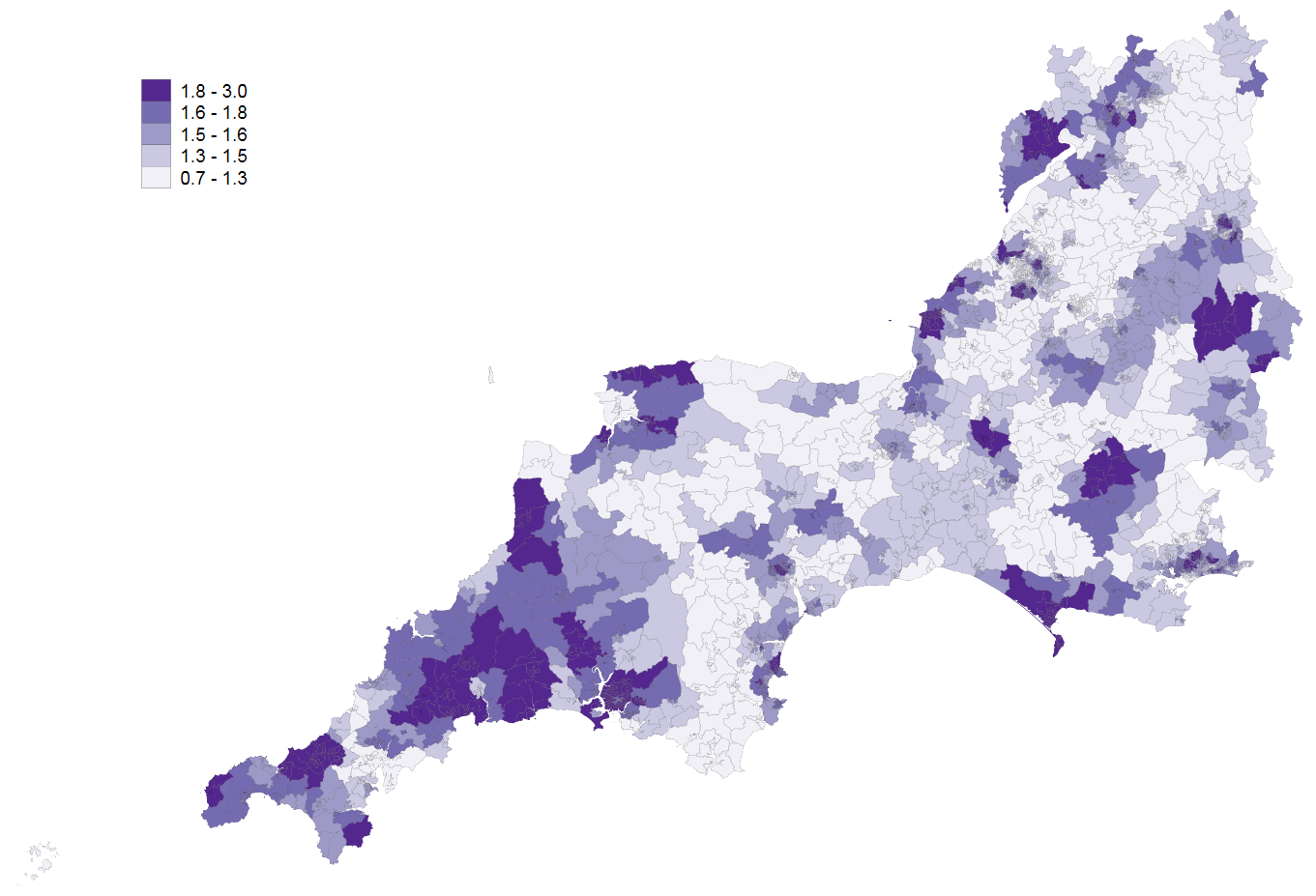
**

**References**

1. Department for Communities and Local Government. The English Indices of Deprivation 2015: Technical Report. Department for Communities and Local Government, 2015.

2. Office for National Statistics. Changes to output areas and super output areas in Englandand Wales, 2001 to 2011. 2012.

3. Bibby P SJ. Developing a new classification of urban and rural areas for policy purposes—the methodology. DEFRA, 2004.

4. Office for National Statistics. Super output area mid-year population estimates for England and Wales, mid-2011 (census based). 2015.

5. Office for National Statistics. Open Geography Portal. 2013.

6. Congdon P. Suicide and parasuicide in London: A small-area study. Urban Stud. 1996;33(1):137-58.

7. Office for National Statistics. Nomis official labour market statistics. 2013.

8. NHS Digital. Prescriptions Dispensed in the Community, Statistics for England - 2006-2016. 2016.

9. Office for National Statistics. Super output area mid-year population estimates for England and Wales, mid-2011 (census based). 2013. [www.ons.gov.uk/ons/publications/rereference-](http://www.ons.gov.uk/ons/publications/rereference-)tables.html?edition=tcm%3A77-285629.

10. NHS Digital. Quality and outcomes framework achievement, prevalence and exceptions data, 2015/16: Frequently asked questions. 2015/16.
